# Supplementary figures and images for: Membrane metalloendopeptidase (MME) is positively correlated with systemic lupus erythematosus and may inhibit the occurrence of breast cancer
Source: PLoS One. 2023 Aug 16;18(8):e0289960. doi: 10.1371/journal.pone.0289960 (PMC10431625; doi:10.1371/journal.pone.0289960)

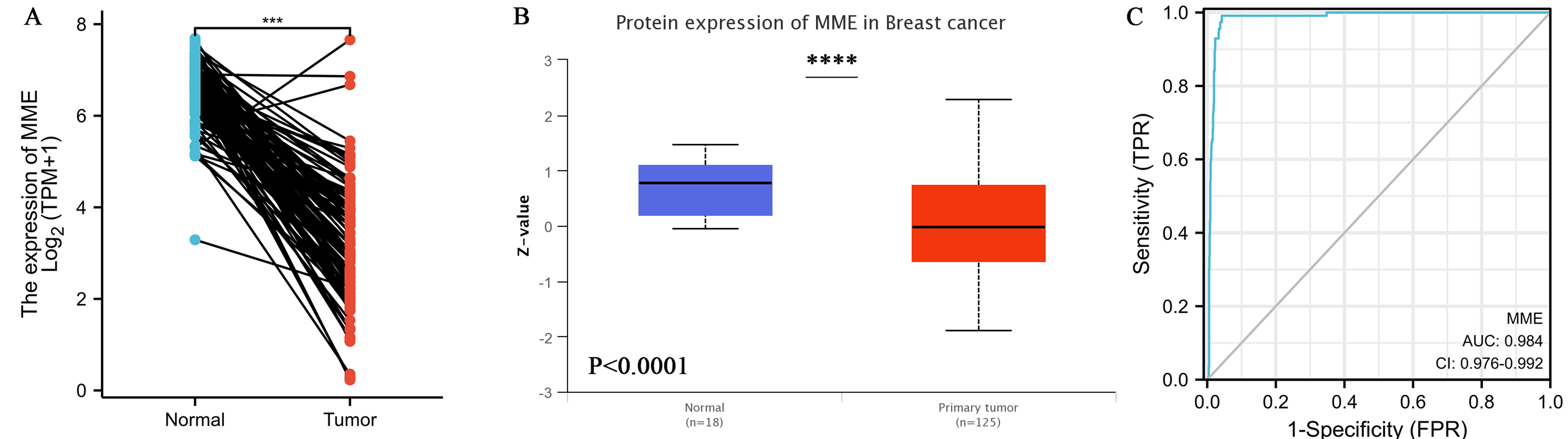

Supplement: S1 Fig — (A) The expression level of MME mRNA between tumor tissue and normal tissue of BRCA. *** P < .001. (B) The expression level of MME total protein between tumor tissue and normal tissue of BRCA. **** P < .0001. (C) ROC analysis was performed to examine the diagnostic value of MME. (TIF) [file pone.0289960.s001.tif]

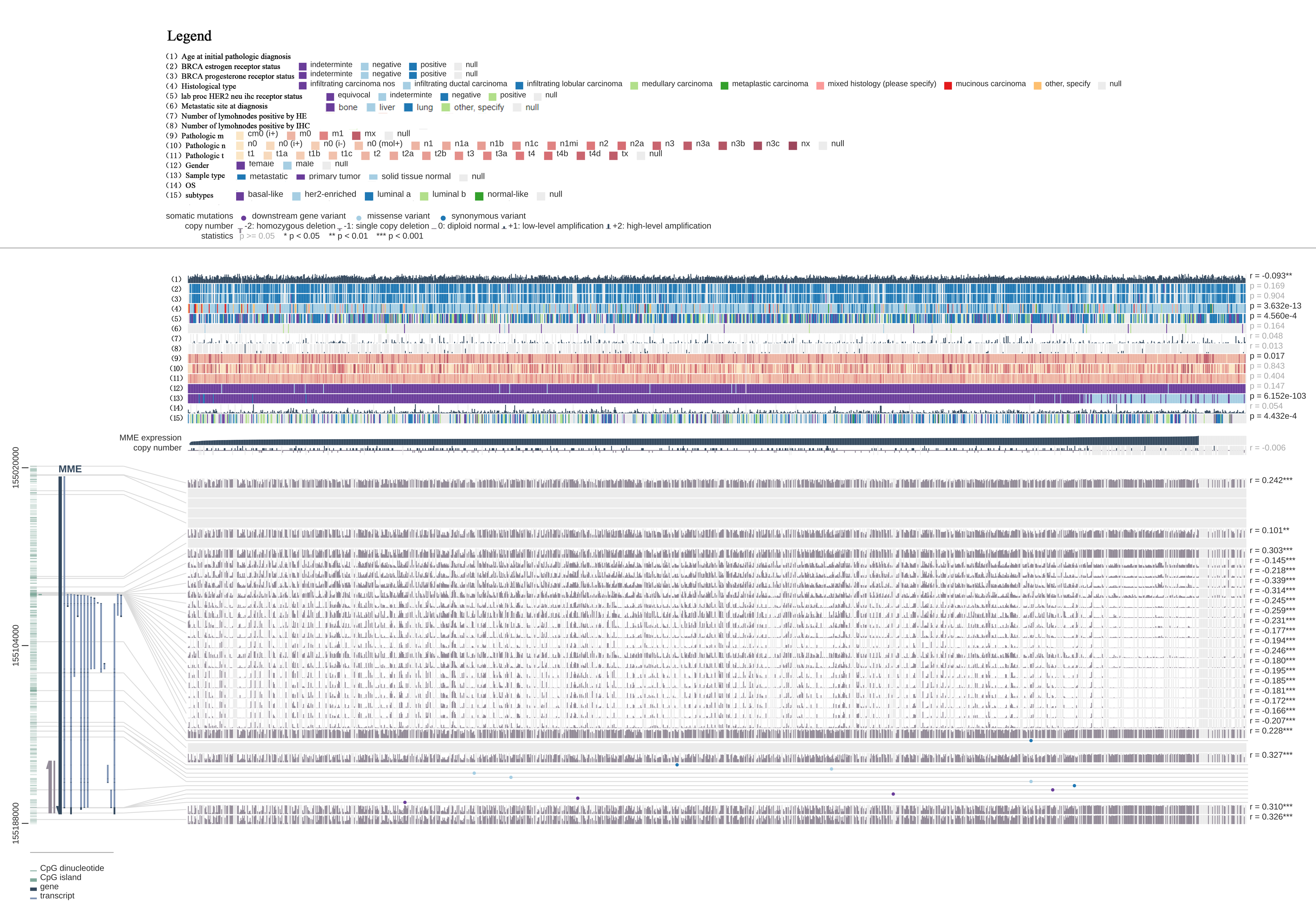

Supplement: S2 Fig — The beta value of methylation, the Benjamini-Hochberg-adjusted P-value and the Pearson correlation coefficients (R) are displayed. (TIF) [file pone.0289960.s002.tif]

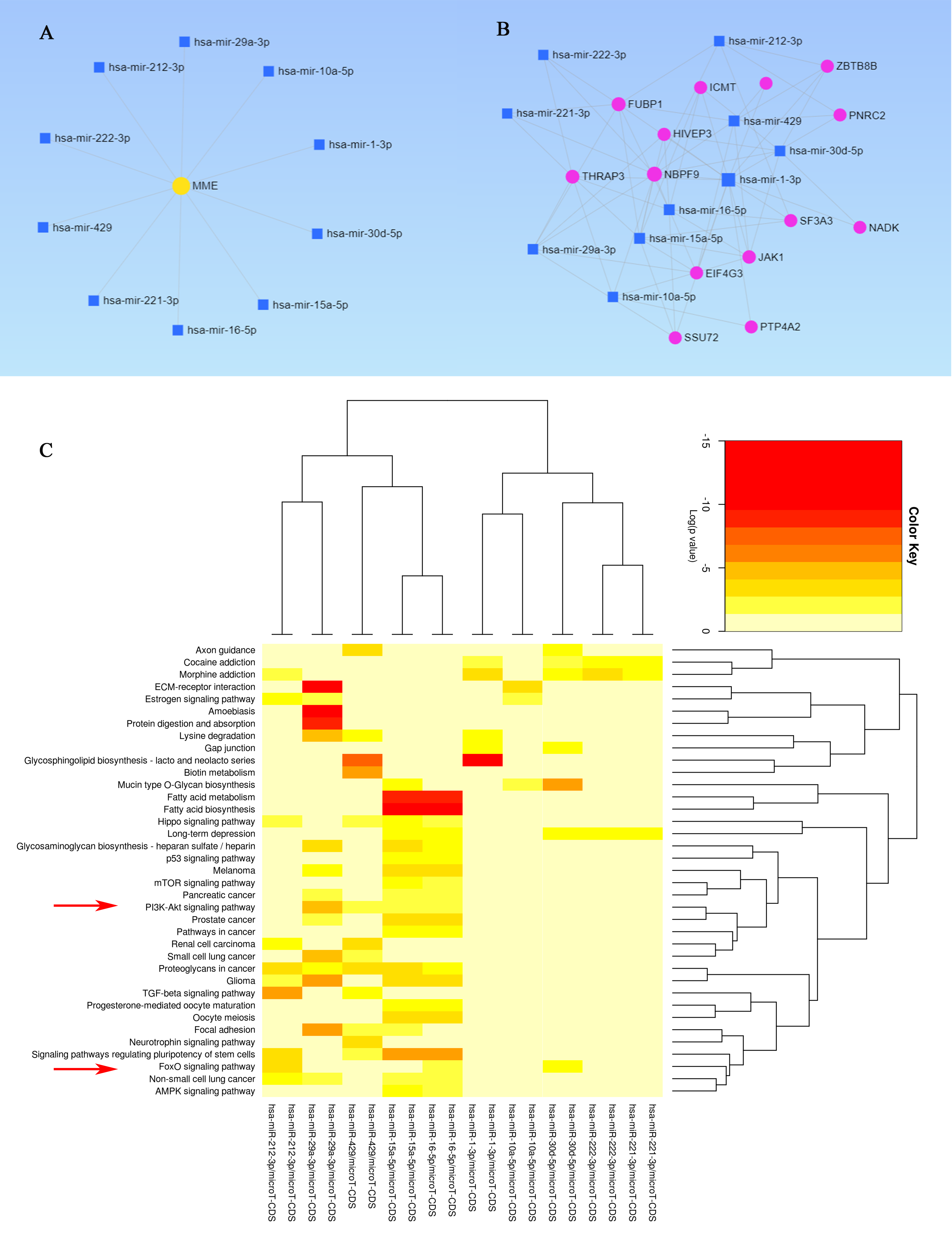

Supplement: S3 Fig — (A) The potential upstream miRNA of MME. (B) The network of MME-related miRNA-circRNA. (C) KEGG enrichment analysis of the potential upstream miRNAs. PI3K/AKT and FOXO signaling pathways were highlighted as the common pathways consistent with the KEGG analysis of MME-related proteins. (TIF) [file pone.0289960.s003.tif]

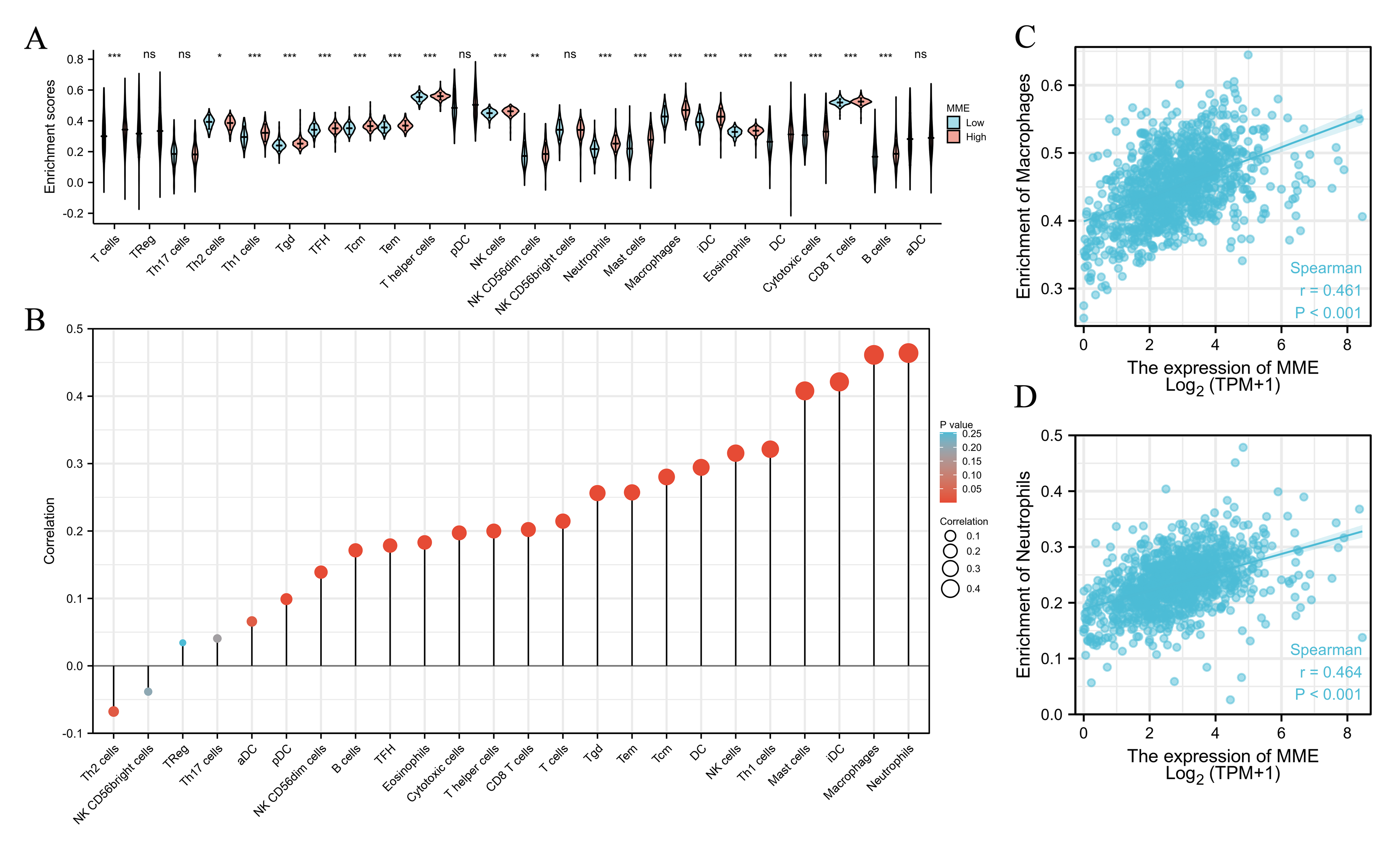

Supplement: S4 Fig — (A) Enrichment scores of immune cells in low and high MME expression group. (B) Lollipop plot illustrated the Spearman’s correlation and p-value of all immune cells. (C) Scatter plot exhibited the Spearman’s correlation and p-value of macrophages. (D) Scatter plot exhibited the Spearman’s correlation and p-value of neutrophils. (TIF) [file pone.0289960.s004.tif]
