# Supplementary material for: Membrane metalloendopeptidase (MME) is positively correlated with systemic lupus erythematosus and may inhibit the occurrence of breast cancer
Source: PLoS One. 2023 Aug 16;18(8):e0289960. doi: 10.1371/journal.pone.0289960 (PMC10431625; doi:10.1371/journal.pone.0289960)
Supplement: S1 Table — (DOCX) [file pone.0289960.s005.docx]

**Table S1** KEGG and GO analysis of MME-related proteins.

|  | **ID** | **Input number** | **Background number** | **P-Value** | **Corrected P-Value** | **Input Gene** |
| --- | --- | --- | --- | --- | --- | --- |
| **KEGG** |  |  |  |  |  |  |
| Protein digestion and absorption | hsa04974 | 6 | 90 | 0.000 | 0.000 | XPNPEP2\|ACE2\|PRCP\|DPP4\|MEP1A\|MEP1B |
| Hematopoietic cell lineage | hsa04640 | 6 | 97 | 0.000 | 0.000 | CD22\|CD34\|CD5\|CD7\|ANPEP\|CD19 |
| Renin-angiotensin system | hsa04614 | 4 | 23 | 0.000 | 0.000 | ACE2\|PRCP\|ANPEP\|ACE |
| B cell receptor signaling pathway | hsa04662 | 4 | 82 | 0.000 | 0.000 | CD22\|LYN\|CD19\|CD79A |
| Primary immunodeficiency | hsa05340 | 2 | 37 | 0.000 | 0.002 | CD19\|CD79A |
| FoxO signaling pathway | hsa04068 | 2 | 132 | 0.002 | 0.016 | PTEN\|BCL6 |
| Cell adhesion molecules (CAMs) | hsa04514 | 2 | 146 | 0.003 | 0.019 | CD22\|CD34 |
| Epstein-Barr virus infection | hsa05169 | 2 | 201 | 0.005 | 0.019 | LYN\|CD19 |
| PI3K-Akt signaling pathway | hsa04151 | 2 | 354 | 0.014 | 0.030 | PTEN\|CD19 |
| Glyoxylate and dicarboxylate metabolism | hsa00630 | 1 | 30 | 0.016 | 0.031 | AGT |
| Alanine, aspartate and glutamate metabolism | hsa00250 | 1 | 36 | 0.019 | 0.034 | AGT |
| Glycine, serine and threonine metabolism | hsa00260 | 1 | 40 | 0.021 | 0.036 | AGT |
| Glutathione metabolism | hsa00480 | 1 | 56 | 0.029 | 0.046 | ANPEP |
| Endometrial cancer | hsa05213 | 1 | 58 | 0.030 | 0.047 | PTEN |
| Long-term depression | hsa04730 | 1 | 60 | 0.031 | 0.048 | LYN |
| Fc epsilon RI signaling pathway | hsa04664 | 1 | 68 | 0.035 | 0.051 | LYN |
| Metabolic pathways | hsa01100 | 3 | 1433 | 0.035 | 0.051 | PTEN\|AGT\|ANPEP |
| Central carbon metabolism in cancer | hsa05230 | 1 | 69 | 0.035 | 0.051 | PTEN |
| Renin secretion | hsa04924 | 1 | 69 | 0.035 | 0.051 | ACE |
| Epithelial cell signaling in Helicobacter pylori infection | hsa05120 | 1 | 70 | 0.036 | 0.052 | LYN |
| p53 signaling pathway | hsa04115 | 1 | 72 | 0.037 | 0.052 | PTEN |
| Melanoma | hsa05218 | 1 | 72 | 0.037 | 0.052 | PTEN |
| Inositol phosphate metabolism | hsa00562 | 1 | 74 | 0.038 | 0.053 | PTEN |
| Glioma | hsa05214 | 1 | 75 | 0.038 | 0.054 | PTEN |
| EGFR tyrosine kinase inhibitor resistance | hsa01521 | 1 | 79 | 0.040 | 0.055 | PTEN |
| Peroxisome | hsa04146 | 1 | 83 | 0.042 | 0.057 | AGT |
| PD-L1 expression and PD-1 checkpoint pathway in cancer | hsa05235 | 1 | 89 | 0.045 | 0.060 | PTEN |
| Hypertrophic cardiomyopathy (HCM) | hsa05410 | 1 | 90 | 0.045 | 0.060 | ACE |
| Small cell lung cancer | hsa05222 | 1 | 93 | 0.047 | 0.062 | PTEN |
| Fc gamma R-mediated phagocytosis | hsa04666 | 1 | 94 | 0.047 | 0.062 | LYN |
| Prostate cancer | hsa05215 | 1 | 97 | 0.049 | 0.064 | PTEN |
| Phosphatidylinositol signaling system | hsa04070 | 1 | 99 | 0.050 | 0.064 | PTEN |
| NF-kappa B signaling pathway | hsa04064 | 1 | 100 | 0.050 | 0.065 | LYN |
| Chagas disease (American trypanosomiasis) | hsa05142 | 1 | 103 | 0.052 | 0.066 | ACE |
| Insulin resistance | hsa04931 | 1 | 108 | 0.054 | 0.068 | PTEN |
| Carbon metabolism | hsa01200 | 1 | 117 | 0.058 | 0.072 | AGT |
| Sphingolipid signaling pathway | hsa04071 | 1 | 119 | 0.059 | 0.073 | PTEN |
| Platelet activation | hsa04611 | 1 | 124 | 0.062 | 0.075 | LYN |
| Autophagy - animal | hsa04140 | 1 | 128 | 0.064 | 0.077 | PTEN |
| Breast cancer | hsa05224 | 1 | 147 | 0.073 | 0.086 | PTEN |
| mTOR signaling pathway | hsa04150 | 1 | 153 | 0.076 | 0.088 | PTEN |
| Cellular senescence | hsa04218 | 1 | 160 | 0.079 | 0.092 | PTEN |
| Hepatocellular carcinoma | hsa05225 | 1 | 168 | 0.083 | 0.095 | PTEN |
| Alzheimer disease | hsa05010 | 1 | 171 | 0.084 | 0.096 | IDE |
| Kaposi sarcoma-associated herpesvirus infection | hsa05167 | 1 | 186 | 0.091 | 0.102 | LYN |
| Transcriptional misregulation in cancer | hsa05202 | 1 | 186 | 0.091 | 0.102 | BCL6 |
| Chemokine signaling pathway | hsa04062 | 1 | 190 | 0.093 | 0.103 | LYN |
| Focal adhesion | hsa04510 | 1 | 199 | 0.097 | 0.107 | PTEN |
| Viral carcinogenesis | hsa05203 | 1 | 201 | 0.098 | 0.108 | LYN |
| Human T-cell leukemia virus 1 infection | hsa05166 | 1 | 219 | 0.106 | 0.116 | PTEN |
| MicroRNAs in cancer | hsa05206 | 1 | 299 | 0.142 | 0.151 | PTEN |
| Human papillomavirus infection | hsa05165 | 1 | 330 | 0.156 | 0.165 | PTEN |
| Pathways in cancer | hsa05200 | 1 | 530 | 0.238 | 0.245 | PTEN |
| **GO** |  |  |  |  |  |  |
| proteolysis | GO:0006508 | 9 | 434 | 0.000 | 0.000 | PRCP\|ACE\|MEP1B\|MME\|IDE\|DPP4\|MEP1A\|XPNPEP2\|ANPEP |
| external side of plasma membrane | GO:0009897 | 8 | 392 | 0.000 | 0.000 | ACE\|CD22\|CD34\|CD79A\|CD5\|ANPEP\|IDE\|CD19 |
| extracellular exosome | GO:0070062 | 11 | 2085 | 0.000 | 0.000 | IDE\|ACE\|CD22\|ANPEP\|ACE2\|LYN\|PRCP\|DPP4\|MEP1A\|XPNPEP2\|CD19 |
| plasma membrane | GO:0005886 | 14 | 4619 | 0.000 | 0.000 | ACE\|CD5\|CD22\|CD34\|CD19\|PTEN\|CD7\|ANPEP\|ACE2\|LYN\|PRCP\|DPP4\|XPNPEP2\|CD79A |
| virus receptor activity | GO:0001618 | 4 | 76 | 0.000 | 0.000 | ACE2\|IDE\|DPP4\|ANPEP |
| peptide catabolic process | GO:0043171 | 3 | 15 | 0.000 | 0.000 | IDE\|ANPEP\|ACE |
| endopeptidase activity | GO:0004175 | 4 | 81 | 0.000 | 0.000 | MME\|ACE2\|IDE\|ACE |
| zinc ion binding | GO:0008270 | 7 | 820 | 0.000 | 0.000 | ACE\|ANPEP\|MME\|ACE2\|IDE\|MEP1A\|MEP1B |
| membrane raft | GO:0045121 | 5 | 237 | 0.000 | 0.000 | ACE2\|LYN\|CD19\|DPP4\|CD79A |
| viral entry into host cell | GO:0046718 | 4 | 89 | 0.000 | 0.000 | ACE2\|IDE\|DPP4\|ANPEP |
| metalloendopeptidase activity | GO:0004222 | 4 | 106 | 0.000 | 0.000 | MME\|IDE\|MEP1A\|MEP1B |
| regulation of cytokine production | GO:0001817 | 3 | 33 | 0.000 | 0.000 | ACE2\|LYN\|BCL6 |
| T cell costimulation | GO:0031295 | 3 | 53 | 0.000 | 0.000 | CD5\|LYN\|DPP4 |
| regulation of immune response | GO:0050776 | 4 | 221 | 0.000 | 0.000 | CD22\|BCL6\|CD34\|CD19 |
| metallopeptidase activity | GO:0008237 | 3 | 63 | 0.000 | 0.000 | ACE2\|ANPEP\|ACE |
| regulation of systemic arterial blood pressure by renin-angiotensin | GO:0003081 | 2 | 5 | 0.000 | 0.000 | ACE2\|ACE |
| protein binding | GO:0005515 | 16 | 11779 | 0.000 | 0.000 | BCL6\|PRCP\|CD5\|IDE\|CD22\|CD34\|CD19\|PTEN\|CD7\|ACE2\|LYN\|AGT\|DPP4\|MEP1A\|MEP1B\|CD79A |
| regulation of blood pressure | GO:0008217 | 3 | 66 | 0.000 | 0.000 | CD34\|ANPEP\|ACE |
| regulation of inflammatory response | GO:0050727 | 3 | 82 | 0.000 | 0.000 | ACE2\|LYN\|BCL6 |
| dipeptidyl-peptidase activity | GO:0008239 | 2 | 10 | 0.000 | 0.000 | PRCP\|DPP4 |
| positive regulation of cellular component movement | GO:0051272 | 2 | 10 | 0.000 | 0.000 | LYN\|BCL6 |
| regulation of B cell receptor signaling pathway | GO:0050855 | 2 | 11 | 0.000 | 0.000 | LYN\|CD19 |
| angiotensin maturation | GO:0002003 | 2 | 12 | 0.000 | 0.000 | ACE2\|ACE |
| extracellular region | GO:0005576 | 7 | 1843 | 0.000 | 0.000 | ACE\|PTEN\|XPNPEP2\|MME\|ACE2\|DPP4\|MEP1B |
| amyloid-beta metabolic process | GO:0050435 | 2 | 13 | 0.000 | 0.000 | IDE\|ACE |
| apical plasma membrane | GO:0016324 | 4 | 354 | 0.000 | 0.001 | PTEN\|DPP4\|CD34\|ACE2 |
| platelet-derived growth factor receptor binding | GO:0005161 | 2 | 15 | 0.000 | 0.001 | PTEN\|LYN |
| B cell receptor signaling pathway | GO:0050853 | 3 | 122 | 0.000 | 0.001 | LYN\|CD19\|CD79A |
| regulation of vasoconstriction | GO:0019229 | 2 | 16 | 0.000 | 0.001 | ACE2\|ACE |
| carboxypeptidase activity | GO:0004180 | 2 | 16 | 0.000 | 0.001 | ACE2\|ACE |
| integral component of plasma membrane | GO:0005887 | 6 | 1380 | 0.000 | 0.001 | CD22\|CD34\|CD5\|MEP1A\|MEP1B\|CD19 |
| regulation of blood vessel diameter | GO:0097746 | 2 | 19 | 0.000 | 0.001 | ACE2\|ACE |
| identical protein binding | GO:0042802 | 6 | 1456 | 0.000 | 0.001 | BCL6\|PTEN\|AGT\|DPP4\|MEP1B\|CD79A |
| metalloaminopeptidase activity | GO:0070006 | 2 | 23 | 0.000 | 0.001 | ANPEP\|XPNPEP2 |
| aminopeptidase activity | GO:0004177 | 2 | 25 | 0.000 | 0.001 | ANPEP\|XPNPEP2 |
| regulation of cell population proliferation | GO:0042127 | 3 | 169 | 0.000 | 0.001 | ACE2\|LYN\|BCL6 |
| extracellular space | GO:0005615 | 6 | 1572 | 0.000 | 0.001 | ACE\|ANPEP\|MME\|ACE2\|IDE\|MEP1A |
| endothelial cell migration | GO:0043542 | 2 | 31 | 0.000 | 0.002 | PTEN\|DPP4 |
| positive regulation of phosphatidylinositol 3-kinase activity | GO:0043552 | 2 | 31 | 0.000 | 0.002 | LYN\|CD19 |
| B cell activation | GO:0042113 | 2 | 32 | 0.000 | 0.002 | CD22\|CD79A |
| cell motility | GO:0048870 | 2 | 36 | 0.000 | 0.002 | PTEN\|CD34 |
| signaling receptor activity | GO:0038023 | 3 | 214 | 0.000 | 0.002 | CD5\|CD7\|ANPEP |
| cell surface | GO:0009986 | 4 | 598 | 0.000 | 0.002 | CD22\|IDE\|DPP4\|ACE2 |
| T cell activation | GO:0042110 | 2 | 44 | 0.000 | 0.003 | CD7\|DPP4 |
| peroxisomal matrix | GO:0005782 | 2 | 52 | 0.000 | 0.004 | IDE\|AGT |
| peptide binding | GO:0042277 | 2 | 61 | 0.000 | 0.005 | IDE\|ANPEP |
| protein targeting to peroxisome | GO:0006625 | 2 | 64 | 0.001 | 0.005 | IDE\|AGT |
| positive regulation of protein binding | GO:0032092 | 2 | 70 | 0.001 | 0.006 | IDE\|ACE |
| negative regulation of ERK1 and ERK2 cascade | GO:0070373 | 2 | 70 | 0.001 | 0.006 | PTEN\|LYN |
| negative regulation of protein phosphorylation | GO:0001933 | 2 | 71 | 0.001 | 0.006 | PTEN\|LYN |
| B cell differentiation | GO:0030183 | 2 | 73 | 0.001 | 0.006 | BCL6\|CD79A |
| adaptive immune response | GO:0002250 | 3 | 347 | 0.001 | 0.006 | LYN\|CD7\|CD79A |
| signaling receptor binding | GO:0005102 | 3 | 353 | 0.001 | 0.007 | CD22\|LYN\|DPP4 |
| positive regulation of neuron differentiation | GO:0045666 | 2 | 82 | 0.001 | 0.007 | PTEN\|BCL6 |
| negative regulation of cell population proliferation | GO:0008285 | 3 | 394 | 0.001 | 0.009 | PTEN\|LYN\|BCL6 |
| cytoplasm | GO:0005737 | 8 | 4624 | 0.001 | 0.011 | CD22\|CD34\|PTEN\|ANPEP\|MME\|ACE2\|LYN\|IDE |
| peroxisome | GO:0005777 | 2 | 108 | 0.001 | 0.012 | IDE\|AGT |
| integral component of membrane | GO:0016021 | 7 | 3643 | 0.002 | 0.013 | ACE\|CD22\|CD7\|ANPEP\|ACE2\|DPP4\|CD79A |
| transmembrane receptor protein tyrosine kinase signaling pathway | GO:0007169 | 2 | 123 | 0.002 | 0.015 | LYN\|CD7 |
| positive regulation of cell population proliferation | GO:0008284 | 3 | 501 | 0.002 | 0.016 | PTEN\|LYN\|DPP4 |
| phosphatidylinositol-3,4,5-trisphosphate 3-phosphatase activity | GO:0016314 | 1 | 5 | 0.003 | 0.019 | PTEN |
| serine-type carboxypeptidase activity | GO:0004185 | 1 | 5 | 0.003 | 0.019 | PRCP |
| receptor-mediated virion attachment to host cell | GO:0046813 | 1 | 5 | 0.003 | 0.019 | ACE2 |
| type 2 immune response | GO:0042092 | 1 | 5 | 0.003 | 0.019 | BCL6 |
| negative regulation of toll-like receptor 2 signaling pathway | GO:0034136 | 1 | 5 | 0.003 | 0.019 | LYN |
| bradykinin catabolic process | GO:0010815 | 1 | 5 | 0.003 | 0.019 | IDE |
| regulation of smooth muscle cell migration | GO:0014910 | 1 | 5 | 0.003 | 0.019 | ACE |
| regulation of germinal center formation | GO:0002634 | 1 | 5 | 0.003 | 0.019 | BCL6 |
| glycosphingolipid binding | GO:0043208 | 1 | 5 | 0.003 | 0.019 | LYN |
| carbohydrate binding | GO:0030246 | 2 | 172 | 0.004 | 0.019 | CD22\|CD34 |
| pyruvate biosynthetic process | GO:0042866 | 1 | 6 | 0.004 | 0.019 | AGT |
| regulation of erythrocyte differentiation | GO:0045646 | 1 | 6 | 0.004 | 0.019 | LYN |
| endothelium development | GO:0003158 | 1 | 6 | 0.004 | 0.019 | CD34 |
| transaminase activity | GO:0008483 | 1 | 6 | 0.004 | 0.019 | AGT |
| antigen receptor-mediated signaling pathway | GO:0050851 | 1 | 6 | 0.004 | 0.019 | CD19 |
| male mating behavior | GO:0060179 | 1 | 6 | 0.004 | 0.019 | PTEN |
| cytosolic proteasome complex | GO:0031597 | 1 | 6 | 0.004 | 0.019 | IDE |
| myelin sheath adaxonal region | GO:0035749 | 1 | 6 | 0.004 | 0.019 | PTEN |
| regulation of blood vessel endothelial cell migration | GO:0043535 | 1 | 6 | 0.004 | 0.019 | PRCP |
| intercellular canaliculus | GO:0046581 | 1 | 6 | 0.004 | 0.019 | DPP4 |
| B cell proliferation involved in immune response | GO:0002322 | 1 | 6 | 0.004 | 0.019 | CD19 |
| regulation of platelet aggregation | GO:0090330 | 1 | 6 | 0.004 | 0.019 | LYN |
| invadopodium membrane | GO:0071438 | 1 | 6 | 0.004 | 0.019 | DPP4 |
| negative regulation of cell aging | GO:0090344 | 1 | 6 | 0.004 | 0.019 | PTEN |
| insulin binding | GO:0043559 | 1 | 6 | 0.004 | 0.019 | IDE |
| immune response-regulating cell surface receptor signaling pathway | GO:0002768 | 1 | 6 | 0.004 | 0.019 | LYN |
| telomerase activity | GO:0003720 | 1 | 6 | 0.004 | 0.019 | PTEN |
| metallodipeptidase activity | GO:0070573 | 1 | 7 | 0.004 | 0.019 | ACE |
| negative regulation of dendritic spine morphogenesis | GO:0061002 | 1 | 7 | 0.004 | 0.019 | PTEN |
| positive regulation of aspartic-type endopeptidase activity involved in amyloid precursor protein catabolic process | GO:1902961 | 1 | 7 | 0.004 | 0.019 | LYN |
| paracrine signaling | GO:0038001 | 1 | 7 | 0.004 | 0.019 | CD34 |
| amyloid-beta clearance by cellular catabolic process | GO:0150094 | 1 | 7 | 0.004 | 0.019 | IDE |
| ubiquitin-dependent protein binding | GO:0140036 | 1 | 7 | 0.004 | 0.019 | IDE |
| transdifferentiation | GO:0060290 | 1 | 7 | 0.004 | 0.019 | CD34 |
| negative regulation of B cell receptor signaling pathway | GO:0050859 | 1 | 7 | 0.004 | 0.019 | CD22 |
| multicellular organismal response to stress | GO:0033555 | 1 | 7 | 0.004 | 0.019 | PTEN |
| leukocyte migration | GO:0050900 | 2 | 192 | 0.004 | 0.019 | LYN\|CD34 |
| protein homodimerization activity | GO:0042803 | 3 | 660 | 0.004 | 0.019 | IDE\|AGT\|DPP4 |
| mitochondrial crista | GO:0030061 | 1 | 8 | 0.005 | 0.019 | LYN |
| glyoxylate metabolic process | GO:0046487 | 1 | 8 | 0.005 | 0.019 | AGT |
| positive regulation of transforming growth factor beta production | GO:0071636 | 1 | 8 | 0.005 | 0.019 | CD34 |
| forebrain morphogenesis | GO:0048853 | 1 | 8 | 0.005 | 0.019 | PTEN |
| regulation of defense response to virus by host | GO:0050691 | 1 | 8 | 0.005 | 0.019 | MME |
| regulation of aerobic respiration | GO:1903715 | 1 | 8 | 0.005 | 0.019 | IDE |
| positive regulation of cardiac muscle contraction | GO:0060452 | 1 | 8 | 0.005 | 0.019 | ACE2 |
| neutrophil mediated immunity | GO:0002446 | 1 | 8 | 0.005 | 0.019 | ACE |
| negative regulation of B cell apoptotic process | GO:0002903 | 1 | 8 | 0.005 | 0.019 | BCL6 |
| neuron-neuron synaptic transmission | GO:0007270 | 1 | 8 | 0.005 | 0.019 | PTEN |
| central nervous system neuron axonogenesis | GO:0021955 | 1 | 8 | 0.005 | 0.019 | PTEN |
| prostate gland growth | GO:0060736 | 1 | 8 | 0.005 | 0.019 | PTEN |
| anaphase-promoting complex binding | GO:0010997 | 1 | 8 | 0.005 | 0.019 | PTEN |
| negative regulation of potassium ion transmembrane transporter activity | GO:1901017 | 1 | 8 | 0.005 | 0.019 | PTEN |
| regulation of transmembrane transporter activity | GO:0022898 | 1 | 8 | 0.005 | 0.019 | ACE2 |
| vascular wound healing | GO:0061042 | 1 | 8 | 0.005 | 0.019 | CD34 |
| germinal center formation | GO:0002467 | 1 | 8 | 0.005 | 0.019 | BCL6 |
| cellular response to insulin-like growth factor stimulus | GO:1990314 | 1 | 8 | 0.005 | 0.019 | PTEN |
| cellular nitrogen compound metabolic process | GO:0034641 | 1 | 9 | 0.005 | 0.019 | AGT |
| antigen processing and presentation of endogenous peptide antigen via MHC class I | GO:0019885 | 1 | 9 | 0.005 | 0.019 | IDE |
| negative regulation of myelination | GO:0031642 | 1 | 9 | 0.005 | 0.019 | PTEN |
| negative regulation of organ growth | GO:0046621 | 1 | 9 | 0.005 | 0.019 | PTEN |
| stem cell proliferation | GO:0072089 | 1 | 9 | 0.005 | 0.019 | CD34 |
| positive regulation of systemic arterial blood pressure | GO:0003084 | 1 | 9 | 0.005 | 0.019 | ACE |
| negative regulation of toll-like receptor 4 signaling pathway | GO:0034144 | 1 | 9 | 0.005 | 0.019 | LYN |
| CD4 receptor binding | GO:0042609 | 1 | 9 | 0.005 | 0.019 | CD22 |
| regulation of immune system process | GO:0002682 | 1 | 9 | 0.005 | 0.019 | BCL6 |
| cell recognition | GO:0008037 | 1 | 9 | 0.005 | 0.019 | CD5 |
| positive regulation of epithelial cell proliferation involved in wound healing | GO:0060054 | 1 | 9 | 0.005 | 0.019 | MME |
| cytokine secretion | GO:0050663 | 1 | 9 | 0.005 | 0.019 | LYN |
| telomere maintenance via recombination | GO:0000722 | 1 | 9 | 0.005 | 0.019 | PTEN |
| negative regulation of gene expression | GO:0010629 | 2 | 210 | 0.005 | 0.019 | CD34\|ACE |
| cellular response to leptin stimulus | GO:0044320 | 1 | 10 | 0.006 | 0.020 | PTEN |
| negative regulation of cell size | GO:0045792 | 1 | 10 | 0.006 | 0.020 | PTEN |
| regulation of mast cell degranulation | GO:0043304 | 1 | 10 | 0.006 | 0.020 | LYN |
| angiogenesis involved in wound healing | GO:0060055 | 1 | 10 | 0.006 | 0.020 | PRCP |
| presynaptic membrane assembly | GO:0097105 | 1 | 10 | 0.006 | 0.020 | PTEN |
| positive regulation of histone deacetylation | GO:0031065 | 1 | 11 | 0.006 | 0.021 | BCL6 |
| wound healing, spreading of epidermal cells | GO:0035313 | 1 | 11 | 0.006 | 0.021 | MME |
| Schmidt-Lanterman incisure | GO:0043220 | 1 | 11 | 0.006 | 0.021 | PTEN |
| negative regulation of axon regeneration | GO:0048681 | 1 | 11 | 0.006 | 0.021 | PTEN |
| negative regulation of excitatory postsynaptic potential | GO:0090394 | 1 | 11 | 0.006 | 0.021 | PTEN |
| regulation of cytokine secretion | GO:0050707 | 1 | 11 | 0.006 | 0.021 | LYN |
| positive regulation of regulatory T cell differentiation | GO:0045591 | 1 | 11 | 0.006 | 0.021 | BCL6 |
| angiogenesis | GO:0001525 | 2 | 238 | 0.007 | 0.021 | PTEN\|ANPEP |
| positive regulation of vasculogenesis | GO:2001214 | 1 | 12 | 0.007 | 0.021 | CD34 |
| cellular response to electrical stimulus | GO:0071257 | 1 | 12 | 0.007 | 0.021 | PTEN |
| maternal behavior | GO:0042711 | 1 | 12 | 0.007 | 0.021 | PTEN |
| synapse maturation | GO:0060074 | 1 | 12 | 0.007 | 0.021 | PTEN |
| locomotor rhythm | GO:0045475 | 1 | 12 | 0.007 | 0.021 | PTEN |
| negative regulation of immune response | GO:0050777 | 1 | 12 | 0.007 | 0.021 | LYN |
| negative regulation of systemic arterial blood pressure | GO:0003085 | 1 | 12 | 0.007 | 0.021 | PRCP |
| locomotory exploration behavior | GO:0035641 | 1 | 13 | 0.007 | 0.021 | DPP4 |
| positive regulation of type I interferon-mediated signaling pathway | GO:0060340 | 1 | 13 | 0.007 | 0.021 | MME |
| dendritic cell differentiation | GO:0097028 | 1 | 13 | 0.007 | 0.021 | LYN |
| chloride ion binding | GO:0031404 | 1 | 13 | 0.007 | 0.021 | ACE |
| positive regulation of cardiac muscle cell apoptotic process | GO:0010666 | 1 | 13 | 0.007 | 0.021 | PTEN |
| mitogen-activated protein kinase kinase binding | GO:0031434 | 1 | 13 | 0.007 | 0.021 | ACE |
| RNA-dependent DNA biosynthetic process | GO:0006278 | 1 | 13 | 0.007 | 0.021 | PTEN |
| negative regulation of blood coagulation | GO:0030195 | 1 | 13 | 0.007 | 0.021 | CD34 |
| immunoglobulin mediated immune response | GO:0016064 | 1 | 13 | 0.007 | 0.021 | CD19 |
| response to arsenic-containing substance | GO:0046685 | 1 | 13 | 0.007 | 0.021 | PTEN |
| glomerular filtration | GO:0003094 | 1 | 13 | 0.007 | 0.021 | CD34 |
| heart contraction | GO:0060047 | 1 | 13 | 0.007 | 0.021 | ACE |
| response to carbohydrate | GO:0009743 | 1 | 13 | 0.007 | 0.021 | LYN |
| regulation of cell adhesion mediated by integrin | GO:0033628 | 1 | 13 | 0.007 | 0.021 | LYN |
| cellular response to DNA damage stimulus | GO:0006974 | 2 | 248 | 0.007 | 0.021 | LYN\|BCL6 |
| intracellular membrane-bounded organelle | GO:0043231 | 3 | 788 | 0.007 | 0.021 | LYN\|AGT\|PRCP |
| negative regulation of phosphatidylinositol 3-kinase signaling | GO:0014067 | 1 | 14 | 0.008 | 0.021 | PTEN |
| cellular response to ethanol | GO:0071361 | 1 | 14 | 0.008 | 0.021 | PTEN |
| positive regulation of ubiquitin protein ligase activity | GO:1904668 | 1 | 14 | 0.008 | 0.021 | PTEN |
| growth hormone receptor signaling pathway via JAK-STAT | GO:0060397 | 1 | 14 | 0.008 | 0.021 | LYN |
| negative regulation of nitric oxide biosynthetic process | GO:0045019 | 1 | 14 | 0.008 | 0.021 | CD34 |
| amyloid-beta clearance | GO:0097242 | 1 | 14 | 0.008 | 0.021 | IDE |
| regulation of release of sequestered calcium ion into cytosol | GO:0051279 | 1 | 14 | 0.008 | 0.021 | LYN |
| response to amyloid-beta | GO:1904645 | 1 | 14 | 0.008 | 0.021 | MME |
| negative regulation of type I interferon-mediated signaling pathway | GO:0060339 | 1 | 14 | 0.008 | 0.021 | MME |
| lysosome | GO:0005764 | 2 | 262 | 0.008 | 0.022 | CD34\|ACE |
| hematopoietic stem cell proliferation | GO:0071425 | 1 | 15 | 0.008 | 0.022 | CD34 |
| cellular protein catabolic process | GO:0044257 | 1 | 15 | 0.008 | 0.022 | IDE |
| basal part of cell | GO:0045178 | 1 | 15 | 0.008 | 0.022 | PRCP |
| regulation of synaptic transmission, GABAergic | GO:0032228 | 1 | 15 | 0.008 | 0.022 | PTEN |
| negative regulation of phagocytosis | GO:0050765 | 1 | 15 | 0.008 | 0.022 | PTEN |
| negative regulation of cardiac muscle cell proliferation | GO:0060044 | 1 | 15 | 0.008 | 0.022 | PTEN |
| positive regulation of glial cell proliferation | GO:0060252 | 1 | 15 | 0.008 | 0.022 | LYN |
| prepulse inhibition | GO:0060134 | 1 | 15 | 0.008 | 0.022 | PTEN |
| regulation of protein kinase B signaling | GO:0051896 | 1 | 16 | 0.009 | 0.023 | PTEN |
| dentate gyrus development | GO:0021542 | 1 | 16 | 0.009 | 0.023 | PTEN |
| endothelial cell proliferation | GO:0001935 | 1 | 16 | 0.009 | 0.023 | CD34 |
| inositol phosphate dephosphorylation | GO:0046855 | 1 | 16 | 0.009 | 0.023 | PTEN |
| hematopoietic stem cell differentiation | GO:0060218 | 1 | 16 | 0.009 | 0.023 | ACE |
| response to drug | GO:0042493 | 2 | 280 | 0.009 | 0.023 | PTEN\|LYN |
| determination of adult lifespan | GO:0008340 | 1 | 17 | 0.009 | 0.023 | IDE |
| positive regulation of ubiquitin-dependent protein catabolic process | GO:2000060 | 1 | 17 | 0.009 | 0.023 | PTEN |
| blood coagulation, intrinsic pathway | GO:0007597 | 1 | 17 | 0.009 | 0.023 | PRCP |
| phosphatidylinositol-3-phosphatase activity | GO:0004438 | 1 | 17 | 0.009 | 0.023 | PTEN |
| long-term synaptic depression | GO:0060292 | 1 | 17 | 0.009 | 0.023 | PTEN |
| negative regulation of B cell proliferation | GO:0030889 | 1 | 17 | 0.009 | 0.023 | LYN |
| cardiac muscle tissue development | GO:0048738 | 1 | 17 | 0.009 | 0.023 | PTEN |
| negative regulation of focal adhesion assembly | GO:0051895 | 1 | 18 | 0.010 | 0.023 | PTEN |
| amino acid binding | GO:0016597 | 1 | 18 | 0.010 | 0.023 | AGT |
| toll-like receptor 4 signaling pathway | GO:0034142 | 1 | 18 | 0.010 | 0.023 | LYN |
| dendritic spine morphogenesis | GO:0060997 | 1 | 18 | 0.010 | 0.023 | PTEN |
| negative regulation of vascular associated smooth muscle cell proliferation | GO:1904706 | 1 | 18 | 0.010 | 0.023 | PTEN |
| response to ATP | GO:0033198 | 1 | 18 | 0.010 | 0.023 | PTEN |
| postsynaptic cytosol | GO:0099524 | 1 | 18 | 0.010 | 0.023 | PTEN |
| regulation of reactive oxygen species metabolic process | GO:2000377 | 1 | 18 | 0.010 | 0.023 | PRCP |
| cellular response to extracellular stimulus | GO:0031668 | 1 | 19 | 0.010 | 0.024 | LYN |
| core promoter sequence-specific DNA binding | GO:0001046 | 1 | 19 | 0.010 | 0.024 | MME |
| negative regulation of cell-matrix adhesion | GO:0001953 | 1 | 19 | 0.010 | 0.024 | BCL6 |
| positive regulation of blood pressure | GO:0045777 | 1 | 19 | 0.010 | 0.024 | ACE |
| erythrocyte development | GO:0048821 | 1 | 19 | 0.010 | 0.024 | BCL6 |
| negative regulation of cellular senescence | GO:2000773 | 1 | 19 | 0.010 | 0.024 | BCL6 |
| ubiquitin-specific protease binding | GO:1990381 | 1 | 20 | 0.011 | 0.025 | PTEN |
| telomerase holoenzyme complex | GO:0005697 | 1 | 20 | 0.011 | 0.025 | PTEN |
| brain morphogenesis | GO:0048854 | 1 | 21 | 0.011 | 0.025 | PTEN |
| negative regulation of axonogenesis | GO:0050771 | 1 | 21 | 0.011 | 0.025 | PTEN |
| sialic acid binding | GO:0033691 | 1 | 21 | 0.011 | 0.025 | CD22 |
| B cell homeostasis | GO:0001782 | 1 | 21 | 0.011 | 0.025 | LYN |
| tissue homeostasis | GO:0001894 | 1 | 21 | 0.011 | 0.025 | CD34 |
| lysosomal membrane | GO:0005765 | 2 | 318 | 0.011 | 0.026 | DPP4\|ANPEP |
| telomerase RNA binding | GO:0070034 | 1 | 22 | 0.012 | 0.026 | PTEN |
| negative regulation of Rho protein signal transduction | GO:0035024 | 1 | 22 | 0.012 | 0.026 | BCL6 |
| lamellipodium membrane | GO:0031258 | 1 | 22 | 0.012 | 0.026 | DPP4 |
| negative regulation of interleukin-2 production | GO:0032703 | 1 | 22 | 0.012 | 0.026 | CD34 |
| mast cell granule | GO:0042629 | 1 | 22 | 0.012 | 0.026 | LYN |
| negative regulation of proteolysis | GO:0045861 | 1 | 22 | 0.012 | 0.026 | IDE |
| arachidonic acid secretion | GO:0050482 | 1 | 24 | 0.013 | 0.028 | ACE |
| posttranscriptional regulation of gene expression | GO:0010608 | 1 | 24 | 0.013 | 0.028 | ACE |
| negative regulation of peptidyl-serine phosphorylation | GO:0033137 | 1 | 25 | 0.013 | 0.029 | PTEN |
| protein catabolic process | GO:0030163 | 1 | 26 | 0.014 | 0.030 | IDE |
| positive regulation of excitatory postsynaptic potential | GO:2000463 | 1 | 26 | 0.014 | 0.030 | PTEN |
| replication fork | GO:0005657 | 1 | 26 | 0.014 | 0.030 | BCL6 |
| ephrin receptor binding | GO:0046875 | 1 | 27 | 0.014 | 0.030 | LYN |
| ionotropic glutamate receptor binding | GO:0035255 | 1 | 27 | 0.014 | 0.030 | PTEN |
| oligodendrocyte development | GO:0014003 | 1 | 27 | 0.014 | 0.030 | LYN |
| positive regulation of reactive oxygen species metabolic process | GO:2000379 | 1 | 27 | 0.014 | 0.030 | ACE2 |
| signal transduction | GO:0007165 | 3 | 1013 | 0.014 | 0.030 | LYN\|CD34\|ANPEP |
| neuronal cell body membrane | GO:0032809 | 1 | 28 | 0.015 | 0.030 | CD22 |
| antigen processing and presentation of peptide antigen via MHC class I | GO:0002474 | 1 | 28 | 0.015 | 0.030 | ACE |
| chemorepellent activity | GO:0045499 | 1 | 28 | 0.015 | 0.030 | DPP4 |
| cellular response to virus | GO:0098586 | 1 | 28 | 0.015 | 0.030 | MME |
| gamma-tubulin binding | GO:0043015 | 1 | 28 | 0.015 | 0.030 | LYN |
| proteolysis involved in cellular protein catabolic process | GO:0051603 | 1 | 28 | 0.015 | 0.030 | IDE |
| positive regulation of release of sequestered calcium ion into cytosol | GO:0051281 | 1 | 29 | 0.015 | 0.031 | CD19 |
| negative regulation of cyclin-dependent protein serine/threonine kinase activity | GO:0045736 | 1 | 29 | 0.015 | 0.031 | PTEN |
| phosphatidylinositol dephosphorylation | GO:0046856 | 1 | 29 | 0.015 | 0.031 | PTEN |
| metallocarboxypeptidase activity | GO:0004181 | 1 | 29 | 0.015 | 0.031 | ACE2 |
| regulation of ERK1 and ERK2 cascade | GO:0070372 | 1 | 29 | 0.015 | 0.031 | LYN |
| mitogen-activated protein kinase binding | GO:0051019 | 1 | 30 | 0.016 | 0.031 | ACE |
| adult behavior | GO:0030534 | 1 | 30 | 0.016 | 0.031 | PTEN |
| platelet-derived growth factor receptor signaling pathway | GO:0048008 | 1 | 30 | 0.016 | 0.031 | PTEN |
| response to axon injury | GO:0048678 | 1 | 30 | 0.016 | 0.031 | LYN |
| protein tyrosine kinase binding | GO:1990782 | 1 | 30 | 0.016 | 0.031 | PTEN |
| inflammatory response | GO:0006954 | 2 | 381 | 0.016 | 0.031 | BCL6\|MEP1B |
| regulation of neuron projection development | GO:0010975 | 1 | 31 | 0.016 | 0.031 | PTEN |
| response to amino acid | GO:0043200 | 1 | 31 | 0.016 | 0.031 | LYN |
| response to zinc ion | GO:0010043 | 1 | 31 | 0.016 | 0.031 | PTEN |
| negative regulation of epithelial to mesenchymal transition | GO:0010719 | 1 | 31 | 0.016 | 0.031 | PTEN |
| behavioral fear response | GO:0001662 | 1 | 32 | 0.017 | 0.032 | DPP4 |
| blood vessel remodeling | GO:0001974 | 1 | 32 | 0.017 | 0.032 | ACE |
| positive regulation of Ras protein signal transduction | GO:0046579 | 1 | 32 | 0.017 | 0.032 | LYN |
| positive regulation of gene expression | GO:0010628 | 2 | 391 | 0.017 | 0.032 | PTEN\|CD34 |
| B cell proliferation | GO:0042100 | 1 | 34 | 0.018 | 0.033 | CD79A |
| lipopolysaccharide-mediated signaling pathway | GO:0031663 | 1 | 34 | 0.018 | 0.033 | LYN |
| regulation of cell differentiation | GO:0045595 | 1 | 34 | 0.018 | 0.033 | BCL6 |
| spermatogenesis | GO:0007283 | 2 | 404 | 0.018 | 0.034 | BCL6\|ACE |
| sequence-specific DNA binding | GO:0043565 | 2 | 405 | 0.018 | 0.034 | MME\|BCL6 |
| phosphoprotein binding | GO:0051219 | 1 | 35 | 0.018 | 0.034 | LYN |
| energy homeostasis | GO:0097009 | 1 | 35 | 0.018 | 0.034 | PRCP |
| cellular response to nerve growth factor stimulus | GO:1990090 | 1 | 35 | 0.018 | 0.034 | PTEN |
| regulation of endocytosis | GO:0030100 | 1 | 35 | 0.018 | 0.034 | CD22 |
| peptidyl-tyrosine autophosphorylation | GO:0038083 | 1 | 36 | 0.019 | 0.034 | LYN |
| negative chemotaxis | GO:0050919 | 1 | 37 | 0.019 | 0.035 | DPP4 |
| phosphatidylinositol 3-kinase signaling | GO:0014065 | 1 | 37 | 0.019 | 0.035 | PTEN |
| protein kinase B signaling | GO:0043491 | 1 | 38 | 0.020 | 0.036 | PTEN |
| negative regulation of Notch signaling pathway | GO:0045746 | 1 | 38 | 0.020 | 0.036 | BCL6 |
| negative regulation of MAP kinase activity | GO:0043407 | 1 | 39 | 0.020 | 0.036 | LYN |
| protein tyrosine/serine/threonine phosphatase activity | GO:0008138 | 1 | 39 | 0.020 | 0.036 | PTEN |
| negative regulation of signaling receptor activity | GO:2000272 | 1 | 39 | 0.020 | 0.036 | PTEN |
| phosphoprotein phosphatase activity | GO:0004721 | 1 | 39 | 0.020 | 0.036 | PTEN |
| positive regulation of interleukin-10 production | GO:0032733 | 1 | 40 | 0.021 | 0.036 | CD34 |
| positive regulation of protein tyrosine kinase activity | GO:0061098 | 1 | 40 | 0.021 | 0.036 | ACE |
| positive regulation of B cell proliferation | GO:0030890 | 1 | 41 | 0.021 | 0.037 | BCL6 |
| negative regulation of G1/S transition of mitotic cell cycle | GO:2000134 | 1 | 41 | 0.021 | 0.037 | PTEN |
| response to cAMP | GO:0051591 | 1 | 41 | 0.021 | 0.037 | AGT |
| toxin transport | GO:1901998 | 1 | 41 | 0.021 | 0.037 | MEP1B |
| response to hormone | GO:0009725 | 1 | 42 | 0.022 | 0.037 | LYN |
| collagen catabolic process | GO:0030574 | 1 | 42 | 0.022 | 0.037 | MME |
| multivesicular body | GO:0005771 | 1 | 42 | 0.022 | 0.037 | CD79A |
| regulation of protein phosphorylation | GO:0001932 | 1 | 44 | 0.023 | 0.039 | LYN |
| scavenger receptor activity | GO:0005044 | 1 | 45 | 0.023 | 0.039 | CD5 |
| response to organic cyclic compound | GO:0014070 | 1 | 45 | 0.023 | 0.039 | LYN |
| long-term synaptic potentiation | GO:0060291 | 1 | 45 | 0.023 | 0.039 | PTEN |
| negative regulation of protein kinase B signaling | GO:0051898 | 1 | 46 | 0.024 | 0.040 | PTEN |
| non-membrane spanning protein tyrosine kinase activity | GO:0004715 | 1 | 46 | 0.024 | 0.040 | LYN |
| response to activity | GO:0014823 | 1 | 47 | 0.024 | 0.040 | PTEN |
| inositol phosphate metabolic process | GO:0043647 | 1 | 47 | 0.024 | 0.040 | PTEN |
| cell adhesion | GO:0007155 | 2 | 478 | 0.025 | 0.041 | CD22\|DPP4 |
| erythrocyte differentiation | GO:0030218 | 1 | 48 | 0.025 | 0.041 | LYN |
| neutrophil degranulation | GO:0043312 | 2 | 482 | 0.025 | 0.041 | PRCP\|ANPEP |
| negative regulation of tumor necrosis factor production | GO:0032720 | 1 | 49 | 0.025 | 0.042 | CD34 |
| negative regulation of neuron death | GO:1901215 | 1 | 50 | 0.026 | 0.042 | CD34 |
| basal plasma membrane | GO:0009925 | 1 | 50 | 0.026 | 0.042 | CD34 |
| social behavior | GO:0035176 | 1 | 51 | 0.026 | 0.043 | PTEN |
| serine-type peptidase activity | GO:0008236 | 1 | 52 | 0.027 | 0.043 | DPP4 |
| cytoplasmic side of plasma membrane | GO:0009898 | 1 | 52 | 0.027 | 0.043 | PTEN |
| regulation of cardiac conduction | GO:1903779 | 1 | 53 | 0.027 | 0.044 | ACE2 |
| pyridoxal phosphate binding | GO:0030170 | 1 | 54 | 0.028 | 0.045 | AGT |
| hemopoiesis | GO:0030097 | 1 | 54 | 0.028 | 0.045 | CD34 |
| cellular response to heat | GO:0034605 | 1 | 55 | 0.028 | 0.045 | LYN |
| chromosome, telomeric region | GO:0000781 | 1 | 55 | 0.028 | 0.045 | PTEN |
| Rho protein signal transduction | GO:0007266 | 1 | 56 | 0.029 | 0.046 | BCL6 |
| protein self-association | GO:0043621 | 1 | 57 | 0.029 | 0.046 | AGT |
| brush border membrane | GO:0031526 | 1 | 58 | 0.030 | 0.047 | ACE2 |
| azurophil granule membrane | GO:0035577 | 1 | 59 | 0.030 | 0.047 | PRCP |
| negative regulation of epithelial cell proliferation | GO:0050680 | 1 | 60 | 0.031 | 0.048 | PTEN |
| learning or memory | GO:0007611 | 1 | 60 | 0.031 | 0.048 | PTEN |
| response to glucocorticoid | GO:0051384 | 1 | 60 | 0.031 | 0.048 | AGT |
| ficolin-1-rich granule membrane | GO:0101003 | 1 | 61 | 0.031 | 0.048 | PRCP |
| negative regulation of neuron projection development | GO:0010977 | 1 | 61 | 0.031 | 0.048 | PTEN |
| protein serine/threonine phosphatase activity | GO:0004722 | 1 | 62 | 0.032 | 0.049 | PTEN |
| endocytic vesicle | GO:0030139 | 1 | 62 | 0.032 | 0.049 | DPP4 |
| synapse assembly | GO:0007416 | 1 | 63 | 0.032 | 0.049 | PTEN |
| apoptotic signaling pathway | GO:0097190 | 1 | 64 | 0.033 | 0.050 | CD5 |
| extrinsic component of cytoplasmic side of plasma membrane | GO:0031234 | 1 | 65 | 0.033 | 0.050 | LYN |
| response to insulin | GO:0032868 | 1 | 65 | 0.033 | 0.050 | LYN |
| extracellular matrix disassembly | GO:0022617 | 1 | 66 | 0.034 | 0.051 | MME |
| chromatin DNA binding | GO:0031490 | 1 | 66 | 0.034 | 0.051 | BCL6 |
| p53 binding | GO:0002039 | 1 | 66 | 0.034 | 0.051 | PTEN |
| response to glucose | GO:0009749 | 1 | 67 | 0.034 | 0.051 | PTEN |
| cellular response to retinoic acid | GO:0071300 | 1 | 67 | 0.034 | 0.051 | LYN |
| collagen binding | GO:0005518 | 1 | 67 | 0.034 | 0.051 | MME |
| positive regulation of tyrosine phosphorylation of STAT protein | GO:0042531 | 1 | 68 | 0.035 | 0.051 | LYN |
| response to nutrient | GO:0007584 | 1 | 68 | 0.035 | 0.051 | PTEN |
| canonical Wnt signaling pathway | GO:0060070 | 1 | 70 | 0.036 | 0.052 | PTEN |
| regulation of GTPase activity | GO:0043087 | 1 | 71 | 0.036 | 0.052 | BCL6 |
| intercellular bridge | GO:0045171 | 1 | 71 | 0.036 | 0.052 | CD34 |
| regulation of insulin secretion | GO:0050796 | 1 | 71 | 0.036 | 0.052 | DPP4 |
| drug binding | GO:0008144 | 1 | 72 | 0.037 | 0.052 | ACE |
| cell differentiation | GO:0030154 | 2 | 596 | 0.037 | 0.052 | LYN\|ANPEP |
| ATP binding | GO:0005524 | 3 | 1463 | 0.037 | 0.053 | PTEN\|LYN\|IDE |
| positive regulation of protein catabolic process | GO:0045732 | 1 | 73 | 0.037 | 0.053 | IDE |
| phosphatidylinositol biosynthetic process | GO:0006661 | 1 | 76 | 0.039 | 0.054 | PTEN |
| protein localization | GO:0008104 | 1 | 77 | 0.039 | 0.055 | BCL6 |
| endoplasmic reticulum-Golgi intermediate compartment | GO:0005793 | 1 | 77 | 0.039 | 0.055 | ANPEP |
| negative regulation of protein binding | GO:0032091 | 1 | 77 | 0.039 | 0.055 | ACE |
| mitochondrial intermembrane space | GO:0005758 | 1 | 78 | 0.039 | 0.055 | LYN |
| protein import into nucleus | GO:0006606 | 1 | 79 | 0.040 | 0.055 | MME |
| amyloid-beta binding | GO:0001540 | 1 | 80 | 0.040 | 0.056 | IDE |
| cell morphogenesis | GO:0000902 | 1 | 81 | 0.041 | 0.057 | BCL6 |
| response to toxic substance | GO:0009636 | 1 | 82 | 0.041 | 0.057 | LYN |
| memory | GO:0007613 | 1 | 82 | 0.041 | 0.057 | PTEN |
| regulation of protein stability | GO:0031647 | 1 | 83 | 0.042 | 0.057 | PTEN |
| locomotory behavior | GO:0007626 | 1 | 85 | 0.043 | 0.058 | PTEN |
| protein phosphatase binding | GO:0019903 | 1 | 86 | 0.043 | 0.058 | CD22 |
| positive regulation of phosphatidylinositol 3-kinase signaling | GO:0014068 | 1 | 86 | 0.043 | 0.058 | LYN |
| PDZ domain binding | GO:0030165 | 1 | 86 | 0.043 | 0.058 | PTEN |
| insulin receptor signaling pathway | GO:0008286 | 1 | 86 | 0.043 | 0.058 | IDE |
| ephrin receptor signaling pathway | GO:0048013 | 1 | 86 | 0.043 | 0.058 | LYN |
| protein tyrosine kinase activity | GO:0004713 | 1 | 89 | 0.045 | 0.060 | LYN |
| kinase activity | GO:0016301 | 1 | 89 | 0.045 | 0.060 | LYN |
| dephosphorylation | GO:0016311 | 1 | 91 | 0.046 | 0.061 | PTEN |
| kidney development | GO:0001822 | 1 | 95 | 0.048 | 0.063 | ACE |
| cell-matrix adhesion | GO:0007160 | 1 | 96 | 0.048 | 0.063 | CD34 |
| perinuclear region of cytoplasm | GO:0048471 | 2 | 697 | 0.049 | 0.064 | LYN\|CD34 |
| cellular response to insulin stimulus | GO:0032869 | 1 | 97 | 0.049 | 0.064 | PTEN |

**Abbreviations:** KEGG: Kyoto Encyclopedia of Genes and Genomes; GO: Gene ontology.
