# Supplementary material for: Membrane metalloendopeptidase (MME) is positively correlated with systemic lupus erythematosus and may inhibit the occurrence of breast cancer
Source: PLoS One. 2023 Aug 16;18(8):e0289960. doi: 10.1371/journal.pone.0289960 (PMC10431625; doi:10.1371/journal.pone.0289960)
Supplement: S2 Table — (DOCX) [file pone.0289960.s006.docx]

**Table S2** The degree and betweenness of the selected miRNA and circRNA in the miRNA-circRNA network**.**

| **RNA** | **Degree** | **Betweenness** |
| --- | --- | --- |
| **miRNA** |  |  |
| hsa-mir-1-3p | 14 | 83.61833 |
| hsa-mir-15a-5p | 9 | 17.79908 |
| hsa-mir-16-5p | 9 | 17.79908 |
| hsa-mir-429 | 7 | 15.40915 |
| hsa-mir-29a-3p | 6 | 6.599959 |
| hsa-mir-30d-5p | 6 | 10.45454 |
| hsa-mir-212-3p | 5 | 6.755873 |
| hsa-mir-10a-5p | 4 | 4.908571 |
| hsa-mir-221-3p | 3 | 0.82771 |
| hsa-mir-222-3p | 3 | 0.82771 |
| **circRNA** |  |  |
| NBPF9 | 10 | 39.8255 |
| FUBP1 | 8 | 21.85485 |
| THRAP3 | 7 | 16.36663 |
| JAK1 | 6 | 11.25493 |
| EIF4G3 | 5 | 5.270653 |
| HIVEP3 | 5 | 5.543031 |
| SSU72 | 4 | 2.481636 |
| ICMT | 4 | 3.093555 |
| ZBTB8B | 4 | 4.784525 |
| SF3A3 | 4 | 3.716336 |
| PNRC2 | 3 | 2.146201 |
| NADK | 2 | 0.764797 |
| HP1BP3 | 2 | 0.606997 |
| PTP4A2 | 2 | 1.290366 |

**Abbreviation:** miRNA: microRNA; circRNA: circular RNA.
