# Supplementary material for: Membrane metalloendopeptidase (MME) is positively correlated with systemic lupus erythematosus and may inhibit the occurrence of breast cancer
Source: PLoS One. 2023 Aug 16;18(8):e0289960. doi: 10.1371/journal.pone.0289960 (PMC10431625; doi:10.1371/journal.pone.0289960)
Supplement: S3 Table — (DOCX) [file pone.0289960.s007.docx]

**Table S3** KEGG and GO analysis of MME-related miRNAs.

|  | **p-value** | **Genes** | **miRNAs** |
| --- | --- | --- | --- |
| **KEGG pathway**  Fatty acid biosynthesis | 0.000 | 4 | 2 |
| ECM-receptor interaction | 0.000 | 20 | 2 |
| Fatty acid metabolism | 0.000 | 7 | 2 |
| Glycosphingolipid biosynthesis - lacto and neolacto series | 0.000 | 5 | 2 |
| Signaling pathways regulating pluripotency of stem cells | 0.000 | 44 | 4 |
| Amoebiasis | 0.000 | 20 | 1 |
| Proteoglycans in cancer | 0.000 | 56 | 5 |
| Glioma | 0.000 | 23 | 4 |
| Morphine addiction | 0.000 | 20 | 5 |
| Long-term depression | 0.000 | 19 | 5 |
| Lysine degradation | 0.000 | 13 | 3 |
| Prostate cancer | 0.000 | 24 | 3 |
| Focal adhesion | 0.000 | 66 | 3 |
| Glycosaminoglycan biosynthesis - heparan sulfate / heparin | 0.000 | 5 | 3 |
| PI3K-Akt signaling pathway | 0.000 | 87 | 4 |
| Melanoma | 0.001 | 21 | 3 |
| Non-small cell lung cancer | 0.001 | 19 | 4 |
| TGF-beta signaling pathway | 0.001 | 17 | 2 |
| Protein digestion and absorption | 0.001 | 23 | 1 |
| Axon guidance | 0.001 | 37 | 2 |
| FoxO signaling pathway | 0.001 | 42 | 3 |
| Hippo signaling pathway | 0.002 | 40 | 4 |
| Cocaine addiction | 0.002 | 13 | 4 |
| Mucin type O-Glycan biosynthesis | 0.003 | 7 | 3 |
| Oocyte meiosis | 0.004 | 17 | 2 |
| Small cell lung cancer | 0.005 | 26 | 2 |
| Renal cell carcinoma | 0.005 | 19 | 2 |
| Pathways in cancer | 0.008 | 38 | 2 |
| Estrogen signaling pathway | 0.012 | 17 | 3 |
| Pancreatic cancer | 0.036 | 13 | 3 |
| mTOR signaling pathway | 0.047 | 12 | 2 |
| p53 signaling pathway | 0.049 | 13 | 2 |
| AMPK signaling pathway | 0.055 | 17 | 2 |
| Gap junction | 0.064 | 13 | 2 |
| Neurotrophin signaling pathway | 0.072 | 22 | 1 |
| Progesterone-mediated oocyte maturation | 0.089 | 15 | 2 |
| Biotin metabolism | 0.111 | 1 | 1 |
| ErbB signaling pathway | 0.126 | 17 | 1 |
| Prolactin signaling pathway | 0.134 | 10 | 1 |
| Ubiquitin mediated proteolysis | 0.185 | 20 | 1 |
| Amphetamine addiction | 0.211 | 14 | 2 |
| MAPK signaling pathway | 0.211 | 32 | 1 |
| Ras signaling pathway | 0.221 | 24 | 1 |
| Chronic myeloid leukemia | 0.232 | 9 | 1 |
| Endometrial cancer | 0.246 | 7 | 1 |
| Glycosaminoglycan biosynthesis - keratan sulfate | 0.254 | 6 | 2 |
| Colorectal cancer | 0.293 | 9 | 2 |
| Wnt signaling pathway | 0.301 | 17 | 2 |
| cGMP-PKG signaling pathway | 0.304 | 21 | 1 |
| Valine, leucine and isoleucine biosynthesis | 0.320 | 2 | 1 |
| Viral carcinogenesis | 0.463 | 20 | 1 |
| Thyroid cancer | 0.491 | 6 | 2 |
| MicroRNAs in cancer | 0.543 | 15 | 1 |
| Platelet activation | 0.596 | 14 | 1 |
| GABAergic synapse | 0.619 | 3 | 1 |
| Insulin signaling pathway | 0.644 | 18 | 2 |
| B cell receptor signaling pathway | 0.728 | 12 | 1 |
| Transcriptional misregulation in cancer | 0.757 | 17 | 1 |
| Choline metabolism in cancer | 0.783 | 12 | 1 |
| Phosphatidylinositol signaling system | 0.800 | 11 | 1 |
| Sphingolipid metabolism | 0.874 | 7 | 1 |
| Thyroid hormone signaling pathway | 0.906 | 9 | 1 |
| Bacterial invasion of epithelial cells | 0.906 | 10 | 1 |
| Arrhythmogenic right ventricular cardiomyopathy (ARVC) | 0.918 | 12 | 1 |
| Circadian rhythm | 0.956 | 4 | 1 |
| Tyrosine metabolism | 0.975 | 2 | 1 |
| Amyotrophic lateral sclerosis (ALS) | 0.975 | 10 | 1 |
| Inositol phosphate metabolism | 0.979 | 9 | 1 |
| Nucleotide excision repair | 0.990 | 3 | 1 |
| 2-Oxocarboxylic acid metabolism | 0.991 | 2 | 1 |
| **GO** |  |  |  |
| protein binding transcription factor activity | 0.000 | 147 | 6 |
| biological_process | 0.000 | 2781 | 6 |
| enzyme binding | 0.000 | 308 | 6 |
| cellular_component | 0.000 | 3257 | 7 |
| cytosol | 0.000 | 585 | 7 |
| epidermal growth factor receptor signaling pathway | 0.000 | 74 | 7 |
| protein complex | 0.000 | 829 | 7 |
| neurotrophin TRK receptor signaling pathway | 0.000 | 95 | 7 |
| gene expression | 0.000 | 154 | 8 |
| Fc-epsilon receptor signaling pathway | 0.000 | 71 | 8 |
| nucleic acid binding transcription factor activity | 0.000 | 278 | 9 |
| molecular_function | 0.000 | 3625 | 10 |
| cellular protein modification process | 0.000 | 674 | 10 |
| biosynthetic process | 0.000 | 956 | 10 |
| cellular nitrogen compound metabolic process | 0.000 | 1129 | 10 |
| ion binding | 0.000 | 1464 | 10 |
| organelle | 0.000 | 2351 | 10 |
| catabolic process | 0.000 | 389 | 6 |
| symbiosis, encompassing mutualism through parasitism | 0.000 | 116 | 6 |
| fibroblast growth factor receptor signaling pathway | 0.000 | 63 | 6 |
| nervous system development | 0.000 | 84 | 6 |
| nucleoplasm | 0.000 | 268 | 7 |
| extracellular matrix disassembly | 0.000 | 26 | 1 |
| blood coagulation | 0.000 | 124 | 8 |
| viral process | 0.000 | 93 | 5 |
| cytoskeletal protein binding | 0.000 | 174 | 5 |
| enzyme regulator activity | 0.000 | 178 | 7 |
| extracellular matrix organization | 0.000 | 44 | 1 |
| endoplasmic reticulum lumen | 0.000 | 33 | 1 |
| collagen catabolic process | 0.000 | 23 | 1 |
| cellular component assembly | 0.000 | 253 | 5 |
| phosphatidylinositol-mediated signaling | 0.000 | 42 | 5 |
| cell death | 0.000 | 188 | 6 |
| platelet activation | 0.000 | 49 | 4 |
| transcription initiation from RNA polymerase II promoter | 0.000 | 49 | 5 |
| axon guidance | 0.000 | 113 | 5 |
| intrinsic apoptotic signaling pathway | 0.000 | 24 | 5 |
| macromolecular complex assembly | 0.000 | 163 | 5 |
| synaptic transmission | 0.000 | 74 | 4 |
| post-translational protein modification | 0.000 | 32 | 2 |
| cellular protein metabolic process | 0.000 | 93 | 5 |
| cell-cell signaling | 0.000 | 100 | 4 |
| membrane organization | 0.000 | 108 | 4 |
| protein complex assembly | 0.000 | 144 | 4 |
| positive regulation of protein insertion into mitochondrial membrane involved in apoptotic signaling pathway | 0.000 | 5 | 2 |
| response to stress | 0.000 | 285 | 4 |
| extracellular matrix structural constituent | 0.000 | 20 | 1 |
| transcription, DNA-templated | 0.000 | 256 | 3 |
| nucleobase-containing compound catabolic process | 0.000 | 143 | 4 |
| small molecule metabolic process | 0.001 | 112 | 2 |
| RNA binding | 0.001 | 186 | 2 |
| transforming growth factor beta receptor signaling pathway | 0.002 | 18 | 2 |
| mitotic cell cycle | 0.002 | 44 | 3 |
| protein binding, bridging | 0.003 | 27 | 2 |
| insulin receptor signaling pathway | 0.005 | 18 | 2 |
| protein ubiquitination | 0.008 | 44 | 2 |
| Notch signaling pathway | 0.010 | 20 | 2 |
| toll-like receptor 10 signaling pathway | 0.018 | 10 | 3 |
| in utero embryonic development | 0.021 | 53 | 2 |
| collagen trimer | 0.022 | 22 | 1 |
| positive regulation of transcription, DNA-templated | 0.022 | 71 | 1 |
| toll-like receptor TLR1:TLR2 signaling pathway | 0.032 | 10 | 2 |
| toll-like receptor TLR6:TLR2 signaling pathway | 0.032 | 10 | 2 |
| homophilic cell adhesion via plasma membrane adhesion molecules | 0.032 | 14 | 2 |
| cell motility | 0.033 | 45 | 1 |
| cellular lipid metabolic process | 0.033 | 16 | 2 |
| protein N-linked glycosylation via asparagine | 0.042 | 13 | 1 |
| Fc-gamma receptor signaling pathway involved in phagocytosis | 0.056 | 12 | 2 |
| extracellular matrix | 0.063 | 35 | 1 |
| collagen fibril organization | 0.064 | 13 | 1 |
| G2/M transition of mitotic cell cycle | 0.065 | 13 | 2 |
| platelet degranulation | 0.080 | 12 | 2 |
| cell junction organization | 0.082 | 18 | 1 |
| JAK-STAT cascade involved in growth hormone signaling pathway | 0.094 | 9 | 2 |
| basement membrane | 0.107 | 19 | 1 |
| microtubule organizing center | 0.127 | 34 | 1 |
| cell adhesion | 0.135 | 51 | 2 |
| transcription corepressor activity | 0.137 | 25 | 1 |
| platelet-derived growth factor binding | 0.138 | 8 | 1 |
| integral component of plasma membrane | 0.140 | 70 | 2 |
| cytoskeleton-dependent intracellular transport | 0.142 | 12 | 2 |
| anatomical structure development | 0.144 | 108 | 2 |
| plus-end-directed microtubule motor activity | 0.155 | 7 | 2 |
| collagen type IV trimer | 0.167 | 6 | 1 |
| negative regulation of translation involved in gene silencing by miRNA | 0.175 | 5 | 1 |
| negative regulation of transcription from RNA polymerase II promoter | 0.185 | 79 | 1 |
| inositol phosphate metabolic process | 0.201 | 6 | 1 |
| kinase activity | 0.202 | 70 | 1 |
| RNA polymerase II core promoter proximal region sequence-specific DNA binding transcription factor activity involved in positive regulation of transcription | 0.209 | 19 | 1 |
| cell junction assembly | 0.210 | 8 | 1 |
| nucleotide-binding oligomerization domain containing signaling pathway | 0.211 | 4 | 1 |
| long-chain fatty-acyl-CoA biosynthetic process | 0.227 | 4 | 2 |
| lung development | 0.231 | 23 | 1 |
| glutamate secretion | 0.262 | 5 | 1 |
| transcription from RNA polymerase II promoter | 0.279 | 31 | 1 |
| chromatin organization | 0.313 | 12 | 1 |
| neuron projection | 0.335 | 29 | 1 |
| ligase activity | 0.359 | 40 | 2 |
| RNA polymerase II core promoter proximal region sequence-specific DNA binding | 0.376 | 20 | 1 |
| Wnt signaling pathway | 0.377 | 24 | 2 |
| glycosaminoglycan metabolic process | 0.377 | 17 | 2 |
| platelet alpha granule lumen | 0.422 | 8 | 2 |
| cyclin-dependent protein kinase holoenzyme complex | 0.448 | 5 | 2 |
| cell cycle arrest | 0.450 | 18 | 1 |
| positive regulation of canonical Wnt signaling pathway | 0.475 | 12 | 2 |
| platelet-derived growth factor receptor binding | 0.476 | 6 | 1 |
| sphingolipid biosynthetic process | 0.495 | 7 | 1 |
| cellular response to amino acid stimulus | 0.502 | 10 | 1 |
| chromatin modification | 0.514 | 13 | 1 |
| regulation of RNA biosynthetic process | 0.541 | 10 | 1 |
| SH3/SH2 adaptor activity | 0.543 | 10 | 1 |
| vesicle-mediated transport | 0.546 | 72 | 1 |
| intracellular receptor signaling pathway | 0.551 | 6 | 1 |
| activation of MAPKK activity | 0.584 | 8 | 1 |
| transport | 0.586 | 198 | 2 |
| cellular response to nerve growth factor stimulus | 0.593 | 5 | 1 |
| beta-1 adrenergic receptor binding | 0.614 | 3 | 1 |
| protein stabilization | 0.687 | 18 | 1 |
| receptor agonist activity | 0.719 | 5 | 2 |
| positive regulation of protein homooligomerization | 0.719 | 3 | 1 |
| positive regulation of signal transduction | 0.737 | 10 | 1 |
| glomerular basement membrane development | 0.790 | 4 | 1 |
| mRNA binding | 0.791 | 9 | 1 |
| platelet-derived growth factor receptor signaling pathway | 0.819 | 7 | 1 |
| frizzled binding | 0.829 | 10 | 2 |
| muscle filament sliding | 0.863 | 6 | 1 |
| cellular response to erythropoietin | 0.869 | 2 | 2 |
| receptor tyrosine kinase binding | 0.874 | 9 | 1 |
| forebrain development | 0.892 | 16 | 1 |
| tongue development | 0.893 | 6 | 1 |
| DNA demethylation | 0.935 | 5 | 1 |
| positive regulation of Ras GTPase activity | 0.938 | 10 | 1 |
| proteinaceous extracellular matrix | 0.942 | 26 | 1 |
| Ras guanyl-nucleotide exchange factor activity | 0.947 | 6 | 1 |
| skin development | 0.950 | 9 | 1 |
| transforming growth factor beta receptor, pathway-specific cytoplasmic mediator activity | 0.961 | 3 | 1 |
| collagen type V trimer | 0.972 | 3 | 1 |
| regulation of cell junction assembly | 0.974 | 3 | 1 |
| methylcytosine dioxygenase activity | 0.974 | 3 | 1 |
| cell-cell junction assembly | 0.981 | 5 | 1 |
| keratan sulfate biosynthetic process | 0.986 | 5 | 1 |
| HECT domain binding | 0.993 | 3 | 1 |
| regulation of oligodendrocyte differentiation | 0.994 | 3 | 1 |
| keratan sulfate metabolic process | 0.994 | 5 | 1 |

**Abbreviations:** KEGG: Kyoto Encyclopedia of Genes and Genomes; GO: Gene ontology.
