# Supplementary material for: Membrane metalloendopeptidase (MME) is positively correlated with systemic lupus erythematosus and may inhibit the occurrence of breast cancer
Source: PLoS One. 2023 Aug 16;18(8):e0289960. doi: 10.1371/journal.pone.0289960 (PMC10431625; doi:10.1371/journal.pone.0289960)
Supplement: S4 Table — (DOCX) [file pone.0289960.s008.docx]

**Table S4** The transcription factors regulating MME.

| **Transcription factor** | **Type** | **Reference (PMID)** |
| --- | --- | --- |
| HOXC6 | Repression | 15637592 |
| MYC | Activation | 11519042 |
| SP1 | Unknown | 14510963 |
| SPI1 | Unknown | 15892171 |

**Abbreviation:** MME: Membrane metalloendopeptidase.
