# Supplementary material for: Membrane metalloendopeptidase (MME) is positively correlated with systemic lupus erythematosus and may inhibit the occurrence of breast cancer
Source: PLoS One. 2023 Aug 16;18(8):e0289960. doi: 10.1371/journal.pone.0289960 (PMC10431625; doi:10.1371/journal.pone.0289960)
Supplement: S5 Table — IC50 of 265 small molecules in 860 cell lines and its corresponding MME mRNA gene expression was obtained from GDSC. (DOCX) [file pone.0289960.s009.docx]

**Table S4** The correlation between GDSC drug sensitivity and MME mRNA expression in pan-cancer. IC50 of 265 small molecules in 860 cell lines and its corresponding MME mRNA gene expression were obtained from GDSC.

| **Drug name** | **Correlation** | **FDR** |
| --- | --- | --- |
| IPA-3 | -0.175 | 0.000 |
| Afatinib | 0.164 | 0.000 |
| Gefitinib | 0.148 | 0.000 |
| Cytarabine | -0.157 | 0.000 |
| XMD8-85 | -0.239 | 0.000 |
| DMOG | -0.141 | 0.000 |
| QL-XI-92 | -0.126 | 0.000 |
| Temsirolimus | -0.145 | 0.000 |
| T0901317 | -0.130 | 0.001 |
| piperlongumine | -0.133 | 0.001 |
| Vinblastine | -0.128 | 0.001 |
| Bleomycin (50 uM) | -0.109 | 0.002 |
| Midostaurin | -0.119 | 0.003 |
| CGP-082996 | -0.201 | 0.004 |
| (5Z)-7-Oxozeaenol | -0.110 | 0.004 |
| BEZ235 | -0.121 | 0.004 |
| TGX221 | -0.167 | 0.005 |
| Embelin | -0.125 | 0.006 |
| Y-39983 | -0.099 | 0.007 |
| BMS-536924 | 0.127 | 0.007 |
| CHIR-99021 | -0.097 | 0.009 |
| Cyclopamine | -0.205 | 0.011 |
| Elesclomol | -0.102 | 0.013 |
| QL-X-138 | -0.089 | 0.014 |
| Obatoclax Mesylate | -0.104 | 0.014 |
| Camptothecin | -0.099 | 0.014 |
| CEP-701 | -0.093 | 0.019 |
| A-770041 | -0.155 | 0.023 |
| HG-6-64-1 | -0.095 | 0.025 |
| SN-38 | -0.089 | 0.027 |
| EKB-569 | 0.085 | 0.028 |
| UNC0638 | -0.077 | 0.029 |
| CMK | -0.189 | 0.030 |
| GSK269962A | -0.092 | 0.034 |
| JW-7-24-1 | -0.076 | 0.036 |
| Parthenolide | -0.180 | 0.037 |
| FR-180204 | 0.093 | 0.040 |
| Pazopanib | -0.097 | 0.041 |
| Shikonin | -0.095 | 0.041 |
| BAY 61-3606 | -0.081 | 0.042 |
| Dasatinib | -0.126 | 0.046 |
| KIN001-055 | 0.097 | 0.048 |
| AZD7762 | -0.078 | 0.050 |
| TW 37 | -0.082 | 0.051 |
| Navitoclax | 0.073 | 0.051 |
| MPS-1-IN-1 | 0.072 | 0.054 |
| CGP-60474 | -0.135 | 0.058 |
| CAY10603 | -0.068 | 0.059 |
| LY317615 | 0.078 | 0.061 |
| AKT inhibitor VIII | 0.080 | 0.064 |
| JW-7-52-1 | -0.143 | 0.065 |
| Mitomycin C | 0.085 | 0.065 |
| AS605240 | 0.075 | 0.068 |
| PI-103 | -0.067 | 0.069 |
| THZ-2-49 | -0.067 | 0.072 |
| CP724714 | 0.083 | 0.072 |
| KIN001-270 | -0.069 | 0.079 |
| TL-1-85 | -0.065 | 0.080 |
| IOX2 | -0.087 | 0.090 |
| BMS-754807 | 0.082 | 0.095 |
| I-BET-762 | -0.060 | 0.096 |
| PAC-1 | -0.068 | 0.099 |
| WH-4-023 | -0.115 | 0.102 |
| Methotrexate | -0.061 | 0.106 |
| JNJ-26854165 | -0.075 | 0.107 |
| Docetaxel | -0.061 | 0.111 |
| Lisitinib | 0.084 | 0.112 |
| CCT007093 | 0.067 | 0.114 |
| PIK-93 | -0.057 | 0.115 |
| Nutlin-3a (-) | -0.071 | 0.115 |
| Lapatinib | 0.098 | 0.119 |
| KIN001-236 | -0.059 | 0.120 |
| 5-Fluorouracil | 0.058 | 0.121 |
| Veliparib | 0.094 | 0.121 |
| Linifanib | 0.070 | 0.131 |
| CH5424802 | 0.075 | 0.135 |
| BMS345541 | 0.054 | 0.140 |
| BX-912 | -0.054 | 0.141 |
| AZD8055 | -0.059 | 0.143 |
| KIN001-244 | -0.055 | 0.143 |
| Dabrafenib | -0.062 | 0.145 |
| TAK-715 | 0.053 | 0.153 |
| UNC1215 | 0.068 | 0.153 |
| Phenformin | -0.053 | 0.160 |
| BX-795 | -0.062 | 0.162 |
| Cetuximab | 0.057 | 0.162 |
| AZD6482 | -0.061 | 0.169 |
| JNK Inhibitor VIII | 0.058 | 0.171 |
| Tubastatin A | -0.050 | 0.178 |
| 681640 | -0.075 | 0.183 |
| KIN001-102 | 0.048 | 0.194 |
| QL-VIII-58 | -0.089 | 0.195 |
| GSK690693 | -0.049 | 0.200 |
| NG-25 | -0.047 | 0.206 |
| Erlotinib | 0.091 | 0.209 |
| NSC-207895 | -0.055 | 0.210 |
| BIX02189 | -0.047 | 0.215 |
| Olaparib | -0.053 | 0.222 |
| CAL-101 | -0.047 | 0.227 |
| HG-5-113-01 | 0.079 | 0.243 |
| OSI-027 | -0.043 | 0.254 |
| Thapsigargin | -0.065 | 0.254 |
| Z-LLNle-CHO | -0.081 | 0.262 |
| VNLG/124 | 0.047 | 0.262 |
| AC220 | 0.051 | 0.273 |
| Bryostatin 1 | -0.055 | 0.277 |
| VX-11e | 0.050 | 0.295 |
| YK 4-279 | -0.055 | 0.295 |
| QL-XII-47 | -0.043 | 0.296 |
| Bortezomib | -0.079 | 0.297 |
| Bleomycin | -0.055 | 0.306 |
| Rapamycin | -0.103 | 0.309 |
| EHT 1864 | 0.061 | 0.311 |
| XMD13-2 | -0.038 | 0.317 |
| PHA-793887 | -0.037 | 0.321 |
| YM155 | -0.050 | 0.324 |
| CUDC-101 | -0.038 | 0.332 |
| Salubrinal | -0.080 | 0.332 |
| RDEA119 | 0.038 | 0.334 |
| PD-0325901 | 0.040 | 0.337 |
| XAV939 | 0.039 | 0.338 |
| FTI-277 | -0.041 | 0.339 |
| AP-24534 | -0.041 | 0.344 |
| CI-1040 | 0.043 | 0.351 |
| CCT018159 | -0.047 | 0.365 |
| GSK429286A | -0.039 | 0.366 |
| Ruxolitinib | 0.046 | 0.366 |
| selumetinib | 0.034 | 0.389 |
| FH535 | -0.043 | 0.390 |
| OSU-03012 | -0.043 | 0.392 |
| Bicalutamide | -0.039 | 0.397 |
| GSK1904529A | -0.039 | 0.416 |
| XMD15-27 | -0.039 | 0.421 |
| Pyrimethamine | -0.087 | 0.421 |
| SGC0946 | 0.043 | 0.429 |
| AUY922 | -0.042 | 0.434 |
| Doxorubicin | -0.048 | 0.444 |
| CX-5461 | -0.030 | 0.447 |
| MLN4924 | -0.043 | 0.457 |
| TG101348 | -0.029 | 0.457 |
| Talazoparib | -0.034 | 0.476 |
| PF-562271 | 0.040 | 0.479 |
| LAQ824 | 0.030 | 0.485 |
| MP470 | -0.030 | 0.511 |
| FMK | 0.044 | 0.525 |
| HG-5-88-01 | 0.076 | 0.531 |
| PF-4708671 | 0.056 | 0.532 |
| Tipifarnib | -0.042 | 0.534 |
| ATRA | 0.033 | 0.535 |
| Belinostat | -0.025 | 0.536 |
| BHG712 | -0.024 | 0.536 |
| PD-0332991 | -0.036 | 0.543 |
| Foretinib | -0.028 | 0.543 |
| NSC-87877 | 0.035 | 0.551 |
| SB590885 | 0.031 | 0.553 |
| JNK-9L | -0.035 | 0.562 |
| YM201636 | -0.023 | 0.569 |
| TAE684 | 0.063 | 0.573 |
| Etoposide | -0.029 | 0.585 |
| SL 0101-1 | 0.041 | 0.601 |
| BMS-708163 | 0.023 | 0.604 |
| Vorinostat | 0.020 | 0.610 |
| SNX-2112 | -0.020 | 0.617 |
| AG-014699 | -0.023 | 0.631 |
| AR-42 | -0.019 | 0.635 |
| PLX4720 | -0.021 | 0.638 |
| VX-702 | 0.070 | 0.640 |
| WZ3105 | 0.018 | 0.642 |
| TL-2-105 | 0.017 | 0.676 |
| PFI-1 | -0.023 | 0.677 |
| MG-132 | -0.041 | 0.683 |
| Nilotinib | 0.024 | 0.684 |
| JQ1 | -0.020 | 0.684 |
| S-Trityl-L-cysteine | -0.037 | 0.685 |
| AS601245 | -0.024 | 0.696 |
| FK866 | 0.015 | 0.704 |
| JQ12 | -0.023 | 0.705 |
| AMG-706 | 0.026 | 0.712 |
| 17-AAG | 0.015 | 0.719 |
| Lenalidomide | 0.030 | 0.725 |
| SB 216763 | -0.019 | 0.732 |
| MS-275 | -0.031 | 0.743 |
| QS11 | -0.023 | 0.746 |
| XMD8-92 | -0.037 | 0.756 |
| KIN001-135 | -0.036 | 0.760 |
| SB 505124 | 0.026 | 0.763 |
| Sorafenib | 0.037 | 0.767 |
| OSI-930 | 0.013 | 0.768 |
| STF-62247 | -0.013 | 0.776 |
| AZ628 | 0.024 | 0.788 |
| GSK1070916 | 0.011 | 0.789 |
| Tivozanib | 0.018 | 0.794 |
| SB52334 | 0.015 | 0.804 |
| Masitinib | -0.011 | 0.805 |
| Ispinesib Mesylate | 0.010 | 0.815 |
| Temozolomide | 0.019 | 0.828 |
| PHA-665752 | 0.035 | 0.829 |
| RO-3306 | -0.010 | 0.836 |
| Axitinib | 0.014 | 0.836 |
| BMS-509744 | -0.023 | 0.837 |
| BIRB 0796 | 0.012 | 0.840 |
| Saracatinib | 0.019 | 0.842 |
| CP466722 | -0.008 | 0.843 |
| KIN001-260 | -0.008 | 0.846 |
| KU-55933 | 0.015 | 0.847 |
| GDC0449 | 0.023 | 0.847 |
| EX-527 | -0.023 | 0.851 |
| NPK76-II-72-1 | 0.007 | 0.862 |
| GDC0941 | 0.012 | 0.862 |
| GSK-650394 | -0.016 | 0.865 |
| Paclitaxel | -0.023 | 0.873 |
| Crizotinib | 0.028 | 0.873 |
| ZM-447439 | -0.009 | 0.873 |
| QL-XII-61 | -0.013 | 0.875 |
| Genentech Cpd 10 | 0.007 | 0.876 |
| GSK2126458 | -0.007 | 0.879 |
| Bexarotene | 0.015 | 0.880 |
| Bosutinib | 0.010 | 0.887 |
| Tamoxifen | 0.020 | 0.893 |
| TPCA-1 | 0.005 | 0.902 |
| ZSTK474 | -0.005 | 0.904 |
| rTRAIL | 0.011 | 0.905 |
| Vinorelbine | -0.008 | 0.914 |
| GW-2580 | 0.039 | 0.916 |
| PD-173074 | -0.019 | 0.920 |
| Gemcitabine | -0.006 | 0.927 |
| A-443654 | -0.022 | 0.928 |
| ZG-10 | -0.008 | 0.930 |
| BI-2536 | -0.012 | 0.931 |
| NU-7441 | -0.009 | 0.934 |
| XL-184 | 0.005 | 0.938 |
| Roscovitine | -0.015 | 0.945 |
| GW 441756 | 0.019 | 0.948 |
| THZ-2-102-1 | -0.003 | 0.949 |
| GW843682X | 0.008 | 0.952 |
| Sunitinib | -0.006 | 0.954 |
| AICAR | 0.002 | 0.961 |
| VX-680 | -0.006 | 0.965 |
| Trametinib | -0.002 | 0.966 |
| GNF-2 | -0.014 | 0.967 |
| AT-7519 | 0.001 | 0.973 |
| KIN001-266 | 0.002 | 0.975 |
| XMD11-85h | 0.006 | 0.980 |
| WZ-1-84 | -0.002 | 0.984 |
| Imatinib | 0.004 | 0.988 |
| MK-2206 | 0.001 | 0.989 |
| Epothilone B | 0.001 | 0.989 |
| LFM-A13 | 0.001 | 0.989 |
| Cisplatin | 0.001 | 0.993 |
| XMD14-99 | 0.000 | 0.995 |
| Zibotentan | -0.002 | 0.996 |

**Abbreviations:** FDR: false discovery rate; GDSC: Genomics of Drug Sensitivity in Cancer; MME: Membrane metalloendopeptidase; IC50, 50%inhibiting concentration.
