# Supplementary material for: Membrane metalloendopeptidase (MME) is positively correlated with systemic lupus erythematosus and may inhibit the occurrence of breast cancer
Source: PLoS One. 2023 Aug 16;18(8):e0289960. doi: 10.1371/journal.pone.0289960 (PMC10431625; doi:10.1371/journal.pone.0289960)
Supplement: Attachment — Submitted filename: Response letter.docx [file pone.0289960.s010.docx]

**Dear Editors and Reviewers:**

Thank you for the letter and for the reviewers’ comments concerning our manuscript, entitled “**Membrane metalloendopeptidase (MME) is positively correlated with systemic lupus erythematosus and may inhibit the occurrence of breast cancer”** (ID: **PONE-D-22-34693**). All of the authors read the peer reviewers’ comments carefully and provided point-by-point response. All of the comments were valuable and very helpful for revising and improving our paper and provided important guiding significance of our research. We have substantially revised the manuscript according to the reviewers' comments. Revised portions are marked in red in the paper. All things considered, the main corrections in the paper and the responses to the reviewers’ comments follow.

**Responses to the reviewer’s comments:**

**Reviewer #1:**

**1. Comment**: The author should demonstrate the interaction between MME and the PI3K/AKT/FOXO signaling pathway in BRCA.

**Response and changes:** Thank you for your advice. We explored the roles of PTEN, BCL6, and CD19, the MME-binding proteins, in the FOXO and PI3K-AKT signaling pathways with the KEGG database in **“Results” section**. Although there is no evidence that MME is directly involved in these two signaling pathways, we believe that MME may regulate them indirectly through the interaction of the abovementioned proteins.

**2. Comment:** Authors should clarify the relationship between MME and all the different types of breast cancer.

**Response and changes**: Thank you for your comments. In **Figure 2G-J**, we added the expression of MME in different histological types of breast cancer and metastases.

**3. Comment:** According to this study, in Conclusion section author need to re-write this sentence “In summary, we identified that MME was negatively related to SLE but positively related to BRCA.”

**Response and changes**: Thank you for pointing out our clerical error; we have now corrected it in **“Conclusion” section**.

**4. Comment:** Figure quality is very bad. Authors should improve.

**Response and changes**: Thank you for pointing this out. We have remade the figures.

**5. Comment:** There are couple of grammatical and spelling mistakes. Minor changes to the manuscript's writing will also help to reinforce the paper.

**Response**: Thank you for pointing out our shortcomings, and we have carefully revised the text.

Thank you for the comments and suggestions.

**Reviewer #2:**

**1. Comment:** Though, we understood, majority of the data were analyzed using primary BC gene sequences and other parameters. It is important to confirm the hypothesis using any dataset from advanced BC or metastatic tissue derived gene sequences.

**Response and changes**: Thank you for your comments. In **Figure 2I and J**, we compare the expression of MME between primary breast cancer and brain, lung, and bone metastases from breast cancer.

**2. Comment:** The second major concern is the manuscript did not address to show the relation between the original function of MME upon the adverse pathology conditions. It Is important that being peptidases, there might be numerous substrates for MME, the manuscript should address the relevance in this manuscript.

**Response**: Thank you for pointing out our shortcomings. MME may act as a tumor suppressor in breast cancer by regulating the PI3K/Akt signaling pathway, especially its downstream FoxO signaling pathway. Moreover, the mechanism responsible for metastasis inhibition by MME seems to be associated with decreased activation of FAK, RhoA, and downstream MEK/ERK, which are involved in tumor migration and metastasis^1^. However, due to the limited current research, the specific mechanism of MME in BRCA still needs further study.

**Changes:** We have further discussed the mechanism of MME in the “**Discussion” section**. However, due to the limited number of current studies, it is difficult to clearly explain the detailed mechanism of MME. It is hoped that our research will arouse the interest of the academic community in MME, which will help us better answer this question.

**3. Comment:** The methodology should be clear and brief. For example ¬ “Additionally, we visited the UALCAN website (<http://ualcan.path.uab.edu/analysis-prot.html>) to explore the protein expression level of MME in the CPTAC dataset [13]”. The website url details may not needed as it is abbreviated, and the detail should be give which dataset and cancer is analyzed
Then, we visited the ROC plotter website (<http://www.rocplot.org/>) to investigate the relationship between MME expression and the response to therapy of breast cancer patients [22].

**Response and changes**: Thank you for your suggestions. We have simplified the “**Method” section** to avoid unnecessary descriptions.

**4. Comment:** The rationale for this analysis DNA methylation, methyltransferases and mismatch repair correlation. further the correlation though significant, not very strong, it is better be removed from this manuscript unless there is strong relevance.

**Response and changes**: Thank you for your suggestion; we have deleted these unimportant results.

**5. Comment:** In addition to the miRNA, TF analysis should give better evidence on the upstream regulator of MME. The manuscript should include the TF analysis.

**Response and changes**: Thank you for your advice. We supplemented this part of the results in the **"Results" section**. With the TRRUST website^2^, we identified HOXC6, MYC, SP1, and SPI1 as transcription factors that regulate the expression of MME (**Table S4**).

Thank you for the comments and suggestions.

We tried our best to improve the manuscript. These changes did not influence the framework of the paper. We did not list all changes here but marked them in red in the revised manuscript.

We earnestly appreciate the editor’s/reviewers’ hard work and hope that the corrections meet with approval.

Once again, thank you very much for the comments and suggestions.

**Reference**

1. Li M, Wang L, Zhan Y, Zeng T, Zhang X, Guan XY, et al. Membrane Metalloendopeptidase (MME) Suppresses Metastasis of Esophageal Squamous Cell Carcinoma (ESCC) by Inhibiting FAK-RhoA Signaling Axis. Am J Pathol. 2019; 189(7):1462-1472.

2. Han H, Cho JW, Lee S, Yun A, Kim H, Bae D, et al. TRRUST v2: an expanded reference database of human and mouse transcriptional regulatory interactions. Nucleic Acids Res. 2018 Jan 4;46(D1):D380-D386.
